# Skeletal muscle myofilament adaptations to aging, disease, and disuse and their effects on whole muscle performance in older adult humans

**DOI:** 10.3389/fphys.2014.00369

**Published:** 2014-09-26

**Authors:** Mark S. Miller, Damien M. Callahan, Michael J. Toth

**Affiliations:** ^1^Department of Kinesiology, School of Public Health and Health Sciences, University of MassachusettsAmherst, MA, USA; ^2^Department of Molecular Physiology and Biophysics, College of Medicine, University of VermontBurlington, VT, USA; ^3^Department of Medicine, College of Medicine, University of VermontBurlington, VT, USA

**Keywords:** myosin, actin, cross-bridge kinetics, single fiber, isometric tension, contractile velocity, sex differences, physical activity

## Abstract

Skeletal muscle contractile function declines with aging, disease, and disuse. *In vivo* muscle contractile function depends on a variety of factors, but force, contractile velocity and power generating capacity ultimately derive from the summed contribution of single muscle fibers. The contractile performance of these fibers are, in turn, dependent upon the isoform and function of myofilament proteins they express, with myosin protein expression and its mechanical and kinetic characteristics playing a predominant role. Alterations in myofilament protein biology, therefore, may contribute to the development of functional limitations and disability in these conditions. Recent studies suggest that these conditions are associated with altered single fiber performance due to decreased expression of myofilament proteins and/or changes in myosin-actin cross-bridge interactions. Furthermore, cellular and myofilament-level adaptations are related to diminished whole muscle and whole body performance. Notably, the effect of these various conditions on myofilament and single fiber function tends to be larger in older women compared to older men, which may partially contribute to their higher rates of disability. To maintain functionality and provide the most appropriate and effective countermeasures to aging, disease, and disuse in both sexes, a more thorough understanding is needed of the contribution of myofilament adaptations to functional disability in older men and women and their contribution to tissue level function and mobility impairment.

## Introduction

Aging, disease, and disuse decrease whole skeletal muscle contractile performance, which reduces an individual's ability to accomplish tasks associated with daily living, eventually leading to physical disability (Guralnik et al., 1995; Janssen et al., 2002; Kortebein et al., 2008). Knowledge of the mechanisms underlying the loss of skeletal muscle performance will aid in the development of suitable exercise and pharmacological countermeasures to forestall or counteract these detrimental changes.

Whole muscle contractile performance has been identified as an important determinant of functional limitations in older adults (Reid and Fielding, 2012). As whole muscle performance is dependent upon the functional character of single muscle fibers (Harridge et al., 1996; D'Antona et al., 2006), which are, in turn, largely determined by the type of myofilament proteins they express and their function (Bottinelli, 2001), alterations in myofilament protein biology may contribute to the development of disability in these conditions. Unfortunately, myofilament properties cannot be discerned from measurements performed at the whole muscle level due to methodological limitations (e.g., estimation of muscle size, muscle architecture), subjective factors (e.g., volitional effort) and the interceding effects of other physiological systems that regulate whole muscle function (e.g., neural, excitation-contraction coupling, connective tissue properties). That is, because myofilaments are the end effectors of muscle contraction, the interceding effects of higher order regulatory factors (i.e., at the cellular, tissue or organ/tissue systems level) can mask variation in myofilament function. Thus, to reliably assess the effects of aging, disease, and disuse on skeletal muscle myofilament biology, a reductionist approach is required, where measurements are obtained at the cellular and molecular levels.

**Figure 1:**
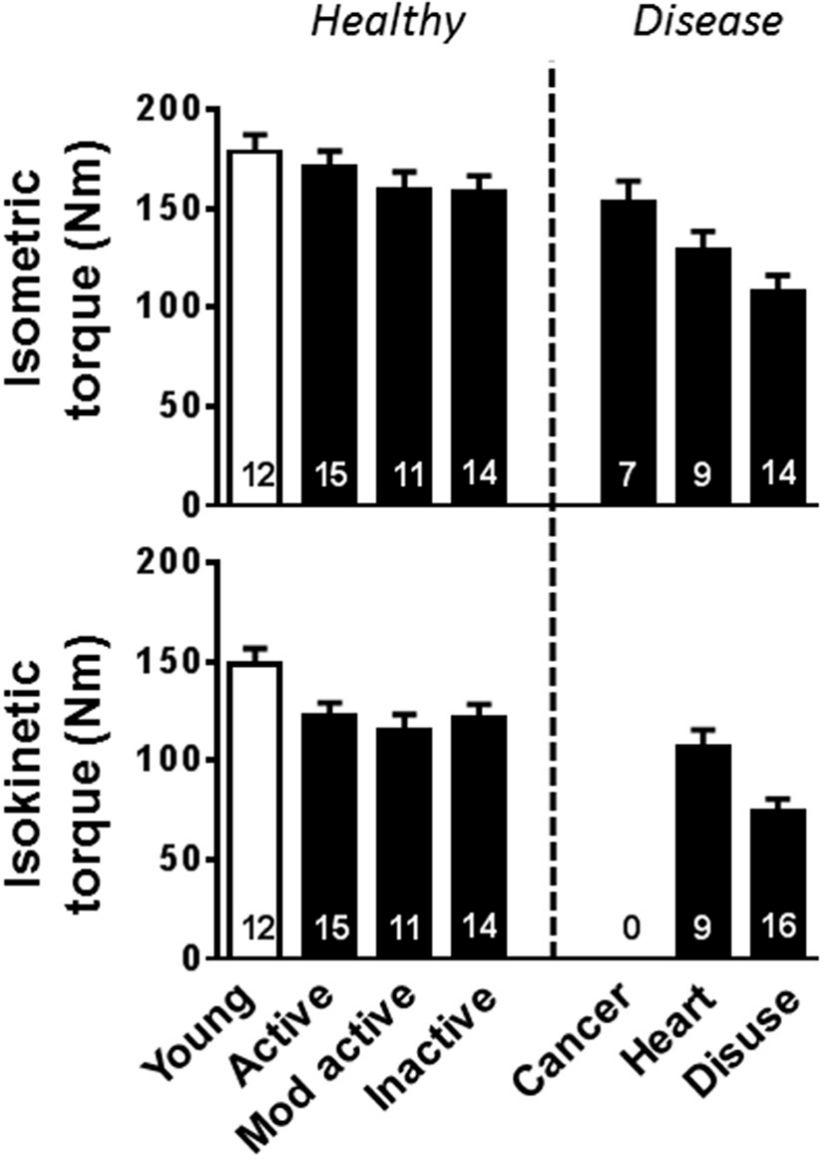
**Whole leg muscle contractile performance changes with aging, disease, and disuse**. Young (open bars) and older (closed bars) are arranged such that whole muscle performance progressively decreases to the right along the x-axis. Volunteers identified as “Young” and “Mod active” are from Miller et al. (2013), “Active” and “Disuse” are from Callahan et al. (2014b), “Inactive” and “Heart” are from Toth et al. (2012), and “Cancer” are from Toth et al. (2013). Isokinetic torque measurements were collected at 60°/s. Mod, moderately; Heart, Heart failure.

This review will focus on human studies that have examined skeletal muscle myofilament structure and function at the cellular and/or molecular levels. As aging, disease, and disuse may alter muscle structure or function by changing muscle quantity [i.e., cross-sectional area (CSA) and/or amount of mass per unit muscle size] or quality (i.e., performance per unit muscle size), we will specifically address these variables in each condition. The review is organized based upon a continuum of whole muscle performance we have observed in our laboratories, which indicates that aging reduces whole muscle performance, both isometric and isokinetic, with progressively larger reductions associated with decreasing activity level, the presence of acute or chronic disease and finally profound, protracted muscle disuse (Figure 1). The reader is referred to several recent reviews of other important aspects of the neuromuscular system that undoubtedly conspire with myofilament adaptations to contribute to functional impairments with aging, disease, and disuse (Raj et al., 2010; Rehn et al., 2012; Reid and Fielding, 2012; Russ et al., 2012; Calvani et al., 2013).

## Myosin-actin cross-bridge (XB) interactions

The formation of the myosin-actin XB is the end effector of muscle contraction. This interaction of two myofilament proteins, myosin and actin, dictate single fiber force and contractile velocity, which can be summarized into simple equations by making several assumptions about the myosin-actin interaction: (1) myosin is either strongly bound to actin producing force or detached from actin producing no force and (2) the XB behaves as a Hookian spring, as detailed (Palmer et al., 2007). In brief, the amount of force that can be produced by a half-sarcomere during isometric contraction can be represented as the number of strongly-bound XBs multiplied by the force generated per XB (F_uni_) (Huxley, 1957; Brenner, 1988). The number of strongly-bound XBs at any point in time is a function of the total number of functional myosin heads (N) multiplied by the fraction of time a strongly-bound XB is formed (t_on_) as a function of total myosin cycle time (t_on_ + t_off_), where t_on_ is the amount of time myosin is strongly bound to actin and t_off_ is the amount of time myosin is detached from actin. Accordingly, the amount of force that can be produced by the half-sarcomere during isometric tension can be written as: N (t_on_/(t_on_ + t_off_)) F_uni_. Notably, t_on_/(t_on_ + t_off_) is commonly called the myosin duty ratio and, although difficult to measure, may be altered under various circumstances, with this effect perhaps best exemplified by differences between the myosin isoforms found in human skeletal muscle (Linari et al., 2004). The force generated per XB may also be altered and can be represented as the elastic stiffness of the XB (k_stiff_) multiplied by the unitary displacement of the myosin power stroke (d_uni_). Contractile velocity is represented most simply as d_uni_ divided by t_on_, in agreement with single fiber findings showing faster velocities with shorter t_on_ (i.e., higher myosin detachment rate) (Piazzesi et al., 2007). Although controversial, recent experimental and modeling work indicates that velocity is also faster with shorter t_off_ (i.e., higher myosin attachment rate) (Hooft et al., 2007; Walcott et al., 2012), potentially via mechanisms that ultimately shorten t_on_ (Walcott et al., 2012). Considered in this context, aging, disease, and/or disuse could alter skeletal muscle force production or velocity by altering the interaction of myosin and actin, which would ultimately affect whole muscle performance and functional capabilities.

What is the relative importance of XB kinetics (e.g., t_on_ and t_off_) vs. XB mechanical properties (e.g., F_uni_ and d_uni_) in determining single fiber function? Single myosin molecule studies comparing myosin heavy chain (MHC) isoforms between [smooth from turkey vs. skeletal from chicken (Guilford et al., 1997)] and within [α vs. β cardiac in rabbit (Palmiter et al., 1999) and rat (Sugiura et al., 1998)] species have shown no differences in F_uni_ or d_uni_, indicating that isoform differences in force and velocity are due primarily to alterations in XB kinetics. In support of XB kinetics being the primary determinants of fiber function, frog single fiber experiments within a single isoform show that F_uni_ and d_uni_ remain consistent over a range of velocities (Piazzesi et al., 2007). In contrast, when looking across fiber types, human single fiber experiments indicate that both XB kinetics and F_uni_ are responsible for the higher forces generated by faster contracting MHC isoforms (Linari et al., 2004). Similarly, single fiber (Brenner et al., 2012) and single molecule (Capitanio et al., 2006) measurements comparing slow isoforms from one species to fast isoforms from another species indicate faster MHC isoforms have higher XB stiffness (k_stiff_), most likely resulting in a higher F_uni_. However, these isoform differences may potentially be due to normal sequence variations as isolated myosin from animal species varying in body size contract at distinctly different velocities (Pellegrino et al., 2003). Overall, these results indicate that XB kinetics play an important role in setting both force and velocity in single muscle fibers, while the role of XB mechanical properties is not clearly defined. Thus, the evaluation of XB kinetics and mechanics as mediators of cellular and tissue level contractile function remains an important area of future study, especially in humans.

Alterations in single fiber force and velocity can also occur via changes in muscle quantity and/or structure. Human skeletal muscle fibers contains three different isoforms (I, IIA, or IIX), with individual fibers expressing either one or multiple isoforms leading to six different fiber types (I, I/IIA, IIA, IIA/IIX, IIX, I/IIA/IIX), with pure MHC I and IIA being the two most prevalent. Parenthetically, an additional embryonic isoform of myosin may be expressed in some physiological/pathophysiological conditions (D'Antona et al., 2003, 2006). A simple shift in the type of MHC isoform expressed in the fiber will produce different tissue level velocity and tension (force per CSA) characteristics, as faster contracting MHCs (I < IIA < IIX) produce more tension (D'Antona et al., 2003, 2006; Pansarasa et al., 2009; Krivickas et al., 2011). Thus, this myofilament variation can alter whole muscle performance (Thorstensson et al., 1977; Ryushi and Fukunaga, 1986; Harridge et al., 1996). In addition to changes in MHC isoform, reducing the amount of myofilament proteins by having smaller fiber CSA, reduced myofibrillar fractional area (e.g., less myofibril area due to an increase in inter-myofibrillar space or other non-contractile elements), or the removal of thick (myosin containing) or thin (actin containing) filaments would decrease single fiber force production. Myofilament ultrastructure plays an important role in setting contractile performance by providing the framework for XB interactions. For instance, at the fiber level, the amount of isometric force produced is equal to the total number of heads interacting in each half-sarcomere (each half-sarcomere must produce identical forces or the sarcomere will change its length). Thus, removal of myosin heads, either from the ends of the thick filament or randomly throughout the thick filament, or a reduction in the number of thin filaments would reduce the number of heads able to interact in a half-sarcomere and result in lower force production. Although fiber CSA is commonly measured in healthy adults as well as during aging, disuse, and disease, changes in myofilament protein content and ultrastructure have not been routinely examined, especially in combination with contractile measurements.

## Aging

Aging reduces whole muscle contractile performance, in part due to a loss of skeletal muscle mass, but also due to reduced muscle quality (Jubrias et al., 1997; Lindle et al., 1997; Lynch et al., 1999; Morse et al., 2005; Delmonico et al., 2009). This age-related diminution in muscle contraction tends to affect dynamic more than isometric function (Lanza et al., 2003; Callahan and Kent-Braun, 2011; Miller et al., 2013), which suggests skeletal muscle properties are altered in a manner that maintains higher forces at slower speeds of movement. Identification of interventions to halt or reverse age-related alterations in skeletal muscle quantity and quality, therefore, can help to maintain physical function and independent living in older adults.

### Muscle quality

Multiple human studies have examined whether aging alters contractile performance at the single fiber and myofilament levels with differing results. Findings are generally concordant across fiber types within an individual study, but age-related adaptations in MHC I and IIA fiber contractile performance (isometric tension and velocity) vary widely across studies, with some showing decreases (Larsson et al., 1997; Frontera et al., 2000, 2008; Krivickas et al., 2001; D'Antona et al., 2003; Ochala et al., 2007; Yu et al., 2007) and others showing that function remains unchanged or even increases (Frontera et al., 2003; Trappe et al., 2003; Korhonen et al., 2006; Reid et al., 2012, 2014; Miller et al., 2013). Notably, longitudinal studies show that single fiber tension and velocity either increase or remain constant (Frontera et al., 2008; Reid et al., 2014), suggesting that older adults do not show continued loss of contractile performance and may, in fact, experience improved myofilament function as a compensatory mechanism to offset whole muscle functional decline.

Although single fiber performance results vary widely, myosin protein function appears to decrease from early (e.g., 20–35 year age range) to the older adult (e.g., 60 years and older). In single fibers, and especially in MHC IIA fibers, aging lowers ATPase (Larsson et al., 1997), slows the time response to changes in fiber length (Ochala et al., 2006, 2007), as well as lengthening t_on_ and potentially t_off_ (Miller et al., 2013), indicating slowing of XB kinetics with age. Using isolated myosin, *in vitro* motility studies show that MHC I actin sliding velocity is decreased (Hook et al., 2001; D'Antona et al., 2003) or unchanged (Canepari et al., 2005) with age, while these same studies agree that MHC IIA actin sliding velocity is unchanged (Hook et al., 2001; D'Antona et al., 2003; Canepari et al., 2005). Thus, in contrast to single fibers, results from studies of isolated myosin suggest that MHC I has the larger aging response of slowing XB kinetics (as slower velocity is indicative of a longer t_on_ and/or t_off_), while MHC IIA is unaffected. These divergent results between these two assays may be related to their experimental conditions as single fiber experiments are performed with their native regulatory protein content and three-dimensional structure using solutions similar to physiologic conditions, while, in order to specifically determine myosin's role in contractile performance, isolated myosin studies remove the myosin from its fiber structure and are typically performed with unregulated actin (Hook et al., 2001; D'Antona et al., 2003; Canepari et al., 2005). Our laboratory (Okada et al., 2008; Miller et al., 2010) and others (Thedinga et al., 1999; D'Antona et al., 2003) have similarly found divergent results when examining myofilament function in isolated myofilaments preparations vs. chemically-skinned single fiber preparations, including when examining genetically-altered animal models (Palmiter et al., 1999; Wang et al., 2013). Based on this evidence, caution must be exercised when interpreting results from these different experimental preparations. Nonetheless, with this caveat in mind, these results collectively suggest that aging generally decreases XB kinetics.

How would slower XB kinetics alter single fiber contractile performance? We would predict that the slowing of XB kinetics, especially longer t_on_ (Miller et al., 2013), should lead to a decrease in contractile velocity. This conclusion is supported by findings of an age-related reduction in contractile velocity that occurs in concert with indications of slower XB kinetics [reduced ATPase activity (Larsson et al., 1997) and longer time response to changes in fiber length (Ochala et al., 2006, 2007)]. At the same time that decreases in XB kinetics reduce velocity, it could have the reciprocal effect of increasing tension, as slower XB kinetics appear to drive an increase in myofilament stiffness, or improved force transmission, that leads to higher isometric tension (Miller et al., 2013). Similarly, slower XB kinetics in older adults occur with an increase in single fiber stiffness, although their overall isometric tension was reduced (Ochala et al., 2006, 2007), which could be explained by reductions in the overall number of XBs secondary to decreased myosin protein expression (D'Antona et al., 2003). Collectively, these studies indicate that the slowing of XB kinetics with age most likely decreases contractile velocity, but may have the paradoxical effect of improving myofilament stiffness and isometric tension.

When findings indicate almost any alteration with age is possible, most noticeable in single fiber contractile performance, this pattern of differences suggests that factors related to the populations studied or the methods employed may explain variation among studies. As the methodology used and experimental conditions are generally standard across studies, variation in the populations studied likely explains the divergent results across studies. One potential reason behind these varying results is differences in physical activity among the populations studied (Figure 1), a factor that is difficult to control for and has commonly not been addressed. Physical activity in older adults is well-known to alter single fiber contractile properties (Trappe et al., 2000, 2001; D'Antona et al., 2003, 2007; Frontera et al., 2003; Parente et al., 2008; Harber et al., 2009; Toth et al., 2012), with higher levels of daily activity or specific exercise training regimens tending to reduce or eliminate age-related differences. Such adaptations are not, however, simply linear throughout the activity spectrum, as immobilization in older individuals has been found to increase velocity (D'Antona et al., 2003, 2007), suggesting that complete loss of weight-bearing activity is characterized by very different adaptations, in keeping with findings from animal studies (Reiser et al., 1987) and muscle unloading due to short duration spaceflight in humans (Widrick et al., 1999). Nonetheless, a modulatory role for physical activity with age would suggest that exercise training may mitigate disability, in part, through a reduction of the deleterious age-related changes in myofilaments.

Another potential confounding factor is differences between men and women in their response to aging. Older women have been found to generate higher isometric tensions in MHC I and IIA fibers (Yu et al., 2007; Miller et al., 2013) and have slower contractile velocities in MHC I (Krivickas et al., 2001; Yu et al., 2007) and IIA (Krivickas et al., 2001) fibers. These results are consistent with the finding of slower XB kinetics being more prominent in both of these fiber types in older women (Miller et al., 2013), as we predict slower kinetics would increase tension and decrease velocity. Sex has also been found to have an effect on force production based upon fiber size, as MHC I and IIA fibers with large CSAs produce less force in women compared to men and MHC I fibers with small CSAs produce more force in women (Frontera et al., 2000). However, other studies have found no statistical differences in the single fiber contractile properties of isometric tension and velocity between older men and women (Trappe et al., 2003; Krivickas et al., 2006), with individual laboratories sometimes showing differing results from study to study (Krivickas et al., 2001, 2006). Such variation among studies may reflect differences in other subject characteristics (e.g., physical activity), the number of fibers being examined [i.e., only 7 MHC IIA fibers in older women (Trappe et al., 2003)], or sample sizes as studies not observing sex differences were smaller (*n* = 16–24) than those observing differences (*n* = 24–38). Notably, a large cross-sectional study (*n* = 71) indicates older mobility-limited females produce higher isometric tension in MHC IIA fibers compared to males (Reid et al., 2012), although a three-year longitudinal study (*n* = 16) from the same laboratory found no sex effects on single fiber performance between healthy and mobility-limited older adults (Reid et al., 2014). Regardless of the reason for variation among studies, these findings raise the possibility that sex differences in myofilament function may contribute to the discrepancies in age-related changes in single fiber performance among studies.

### Muscle quantity/structure

In general, studies using individual skinned fibers find no change in MHC I and IIA CSA with age (Frontera et al., 2000, 2003, 2008; Ochala et al., 2007; Hvid et al., 2010; Reid et al., 2012, 2014), although some have found decreases in MHC I (D'Antona et al., 2003), IIA (Larsson et al., 1997), or both (Korhonen et al., 2006). CSA also decreases with inactivity in both fiber types (D'Antona et al., 2003). Moreover, CSA has either no (Frontera et al., 2000; Reid et al., 2012, 2014) or varied response to aging in men vs. women, with older men having larger CSAs than young in MHC I fibers (Miller et al., 2013) and older men (Yu et al., 2007) or women (Trappe et al., 2003; Miller et al., 2013) having smaller CSAs than their young counterparts. An advantage of CSA measurements in single skinned fibers is that they are typically performed at the experimental sarcomere length (usually ~2.50–2.75 μm), providing a consistent standard across fiber types and age groups. However, one caveat to the skinned fiber preparation is that it undergoes swelling, which may complicate age comparisons. When CSA measurements are determined from both skinned fibers used for contractile performance and fresh frozen preparations via histochemistry within the same group of volunteers, the two techniques can produce different results, with histochemistry results finding no change in MHC I fibers with age and decreasing in MHC IIA with age (Korhonen et al., 2006; Miller et al., 2013; Callahan et al., 2014a). However, recent studies suggest that age-related differences in skinned human single muscle fibers are comparable to differences observed with fresh frozen preparations (Hvid et al., 2011). Parenthetically, studies in genetic animal models (Blaauw et al., 2009), where muscle fiber protein content is dramatically altered (e.g., 50% hypertrophy in 3 weeks due to activation of Akt signaling) showed disparate CSA results between skinned and fresh frozen preparations, suggesting that variation in the degree of swelling may occur with alterations in fiber protein content, urging caution with this approach. Studies examining whole vastus lateralis muscle indicate that the age-related reduction in muscle size is due to fibers being lost and a decrease in CSA, especially in MHC II fibers (Lexell et al., 1988; Lexell and Downham, 1991; Lexell, 1995). In contrast, a recent metaanalysis, which did not include skinned fiber results, indicates CSA is unchanged with age, but is larger with higher physical activity levels (Gouzi et al., 2013). Thus, the variation in CSA with age between the various studies may be due to other confounding factors. Overall, these results indicate that CSA with age either remains unchanged or decreases in MHC II fibers, with alterations possibly affected by sex. Despite potential changes in CSA, recent data indicates that the amount of myofilaments as well as their general structure and stoichiometry is unaltered with age or sex (Callahan et al., 2014a). However, studies do indicate age-related changes in post-translational modifications, such as decreased phosphorylation of the fast isoform of the regulatory myosin light chain (Gelfi et al., 2006; Miller et al., 2013), and in gene expression of protein isoforms, such as an increased gene expression of tropomyosin 2 (Roth et al., 2002). These types of alterations have not been commonly examined in human, but may play a role in functional adaptations as they can affect XB performance.

As the MHC isoform distribution and content can alter contractile performance, several studies have examined the age-related changes in these properties. MHC isoform distribution remained unchanged in longitudinal studies (Frontera et al., 2008) and when physical activity was matched between young and older volunteers (D'Antona et al., 2007; Miller et al., 2013). However, activity level is generally thought to modify MHC isoform distribution, with lower activity causing a shift toward a faster phenotype, or an increase in MHC IIX (D'Antona et al., 2003, 2007), and higher activity causing a shift toward a slower phenotype, or an increase in MHC I (Korhonen et al., 2006; D'Antona et al., 2007). MHC content also remained unchanged in studies matching for physical activity levels (Trappe et al., 2003; Miller et al., 2013) and decreases with reductions in physical activity and immobilization (D'Antona et al., 2003). Altogether, these studies indicate that the amount or types of MHC are not altered by age, but can be changed in older adults based upon their habitual level of exercise.

## Heart failure

Heart failure represents the final common pathway for most chronic cardiovascular disease, and is characterized by an inability of the heart to pump blood to meet the metabolic demands of the body, or that it can only do so at elevated filling pressures. Accordingly, exercise intolerance is the hallmark symptom of the disorder, leading to high rates of disability (Pinsky et al., 1990). Although reduced cardiac contractile performance undoubtedly contributes to reduced functional capacity, changes in skeletal muscle physiology, including muscle atrophy, weakness and reduced oxidative capacity are well-accepted contributors (Zizola and Schulze, 2013). A more complete understanding of the mechanisms causing muscle dysfunction, therefore, is important for identifying appropriate countermeasures to maintain or improve the whole muscle performance and, in turn, reducing disability in these patients.

### Muscle quantity/structure

Skeletal muscle atrophy in patients with end-stage heart failure has been recognized for decades (Pittman and Cohen, 1964) and studies have identified reductions in single muscle fiber size in patients, especially in MHC IIA fibers (Mancini et al., 1989; Massie et al., 1996; Szentesi et al., 2005), although others have shown that CSA remains unchanged in stable, ambulatory heart failure patients (Sullivan et al., 1990; Schaufelberger et al., 1995, 1997; Miller et al., 2009). Prior results of reduced CSA may be partially explained by muscle disuse-related atrophy, as few studies have controlled for this confounding factor, or because patients were studied shortly following hospitalization. Interestingly, human studies (Toth et al., 2005; Miller et al., 2009), along with animal models (Van Hees et al., 2007), have shown a phenotype of reduced myosin content/functional XB number at the whole tissue and single fiber levels per unit protein or fiber CSA using biochemical (gel electrophoresis) and mechanical (rigor stiffness) measurements, and this loss is observed in patients even when compared to activity-matched controls (Miller et al., 2009). These studies have also suggested that there was no specific reduction in actin protein or thin filament content with heart failure (Miller et al., 2009). At the ultrastructural level, myosin loss could present as a decreased number of thick filaments, shortened thick filaments or a combination of both adaptations. However, no change in either thick-to-thin filament stoichiometry or thick filament length has been found (Miller et al., 2009). Based on this evidence, we hypothesized that myosin was lost at random throughout the thick filament, which would agree with studies that indicate remodeling of the thick filaments takes place by removing and replacing myosin molecules at random throughout the length of the filament (Wenderoth and Eisenberg, 1987; Franchi et al., 1990). Further confirmation of this pattern of thick filament adaption in heart failure will be difficult, as the resolution of current microscopic approaches is insufficient to rigorously test this hypothesis. Nonetheless, the loss of myosin from muscle fibers has clear functional relevance (discussed below).

On a more macroscopic level, heart failure has long been associated with a shift in skeletal muscle fiber type toward a more fast-twitch phenotype (Mancini et al., 1989; Vescovo et al., 1996; Sullivan et al., 1997; Toth et al., 2005). A shift in MHC isoform distribution of this nature could contribute to reduced exercise tolerance given the differing oxidative potential of MHC I and II fibers and this has been demonstrated in patients (Vescovo et al., 1998). However, this pattern of isoform distribution is also emblematic of muscle disuse (Narici and De Boer, 2011) and heart failure patients expend about half as many calories in volitional activity as healthy controls (Toth et al., 1997), suggesting that this adaptation might be due to inactivity accompanying the disease, rather than the disease process itself. This appears to be the case, as we have recently found no alteration in muscle MHC protein isoform expression in patients when compared to activity-matched controls (Miller et al., 2009), and others have shown no differences in MHC isoform expression between patients and controls matched for peak aerobic fitness (Mettauer et al., 2001). There is limited information regarding shifts in isoform expression of other myofilament proteins. One study showed a shift in myosin light chain and troponin T, I, and C toward a slow-twitch isoform distribution in diaphragm muscle in heart failure patients commensurate with a shift in MHC expression toward the MHC I isoform (Tikunov et al., 1996). However, unlike peripheral skeletal muscle, the diaphragm muscle undergoes different functional adaptations (i.e., increased work due to increased pulmonary resistance vs. muscle disuse in peripheral skeletal muscle). In peripheral skeletal muscle, myosin light chain isoform distributions in MHC IIA single fibers were found to show a trend toward greater expression of MLC 1f in heart failure patients (Miller et al., 2010), but this was not associated with differences in single fiber function. Although an indirect measure, an alteration in Ca^2+^ sensitivity was noted in MHC IIA fibers, which could reflect adaptations in actin-associated regulatory proteins, albeit this was not directly measured.

### Muscle quality

Independent of variation in muscle size, numerous studies have suggested that there are reductions in skeletal muscle contractile function at the whole muscle [i.e., tissue level (Harrington et al., 1997; Toth et al., 2010)], suggesting decreased intrinsic function of muscle that may be due to myofilament deficiencies, impaired excitation-contraction coupling (Reiken et al., 2003) or a combination of the two. Studies in humans and animal models over the past 5–10 years have shed light on the possible involvement of myofilaments in these contractile deficits.

The most prominent quantitative/structural change that would be predicted to contribute to strength losses per unit muscle size is a loss of MHC, which should decrease the total number of functional myosin heads, causing a reduction in single fiber isometric tension. Indeed, recent studies have found reduced tension in both in MHC I and IIA fibers from heart failure patients (Szentesi et al., 2005) and in experimental models (Van Hees et al., 2007). However, studies from our laboratory, in which heart failure patients were matched for both age and physical activity level, unlike prior studies (Szentesi et al., 2005), found no diminution in single fiber tension (Miller et al., 2010), arguing that prior results of reduced tension with heart failure were explained more by muscle disuse and/or aging than by heart failure *per se*. The question then becomes: how is tension maintained in the face of a reduction in the overall amount of myosin/functional XBs? We found that XB kinetics were slowed, manifested most prominently as a longer t_on_, in MHC I and IIA fibers, in patients compared to controls (Miller et al., 2010), a result that has also been observed in animal models of heart failure (Van Hees et al., 2007). This reduction in XB kinetics, if not compensated for by an increase in t_off_, would lead to an increase in residence time of myosin in the strongly-bound state and, in turn, more myosin heads bound to actin as a function of total myosin cycle time. This is indeed the case, as we found no evidence for a reduction in strongly-bound myosin-actin XBs during maximal Ca^2+^ activation in heart failure patients using different techniques (Miller et al., 2009, 2010). Thus, adaptations in myosin kinetic properties compensate for the loss of myosin protein content to preserve isometric contractile strength. In fact, building off of our findings from myofilament ultrastructural measures that suggested a random loss of myosin heads along the length of the thick filament detailed above (Miller et al., 2009), we recently reported the results of modeling studies which suggest that this pattern of myosin loss is associated with an increase in myosin attachment time (Tanner et al., 2014). Thus, we hypothesize that a random removal of myosin from the thick filament alters the load imparted to a strongly-bound XB causing a lengthening of t_on_, leading to a higher duty ratio and higher tension generation.

Although single muscle fiber tension is maintained, reductions in myosin kinetics in heart failure patients may not be without functional consequences. We would predict that reduced XB kinetics, and increased t_on_ specifically, would reduce contractile velocity (Miller and Toth, 2013). Indeed, recent studies from our laboratory show that this is the case in MHC IIA fibers, where lower XB kinetics were associated with decreased single fiber shortening velocity and, in turn, power output (Callahan et al., 2014b). Thus, while we found no evidence for decrements in single fiber tension that would scale to the tissue level to explain reduced isometric force production (Harrington et al., 1997; Toth et al., 2010), reduced myosin-actin XB kinetics may explain reduced muscle power output during dynamic contractile conditions (Toth et al., 2010). This is noteworthy, as reductions in lower-extremity power output are a primary determinant of physical disability (Reid and Fielding, 2012). Thus, alterations in myofilament properties to yield a reduction in the overall capacity for muscle power output during dynamic activities would mean that any activity would be performed at greater percentage of the muscle's physiological capacity and, in turn, may be more fatiguing. In this context, myofilament adaptations may partially contribute to the subjective sensation of exercise intolerance present in heart failure patients, the primary symptom of the disease.

## Cancer

Cancer has well-known effects on skeletal muscle that have been the subject of intense investigation for decades. The most notable effects of cancer are to reduce overall muscle mass secondary to the profound wasting that occurs with the disease [i.e., cancer cachexia (Fearon et al., 2012)], which has, by far, been studied most extensively. However, atrophy is usually confined to certain cancers (e.g., lung, pancreatic, head, and neck) or the later stages of the disease. In contrast, estimates suggest that up to 90% of patients experience subjective functional deficits, manifested most notably as cancer-related fatigue (Cella et al., 2001). Subjective fatigue is a complex psycho-physiological construct, but likely has some roots in reductions in physiological capacity and a recent study suggests that this may relate, in part, to alterations in myofilament biology (Toth et al., 2013).

### Muscle quantity/structure

Cancer is primarily thought to influence skeletal muscle through its ability to promote muscle atrophy, and has recently been reviewed in detail (Fearon et al., 2012). Despite the well-known atrophic effects of cancer, there are surprisingly few studies that have examined its effects on single muscle fiber size in humans (Weber et al., 2009), although recent work confirms that the atrophy observed at the tissue level extends to the cellular level (Toth et al., 2013). Although profound reductions in the overall amount of contractile tissue occur, recent ultrastructural studies suggest that atrophy is characterized by stoichiometric reductions in the myofilament content relative to fiber CSA (Toth et al., 2013). In contrast to these anatomic measurements, studies that have evaluated myofilament protein content using biochemical approaches in both pre-clinical models (Acharyya et al., 2004) and patients (Eley et al., 2008) have suggested that atrophy in cancer is associated with a selective loss of the contractile protein myosin, with relative preservation of other contractile proteins. If present, one would predict that such an adaptation would diminish muscle contractile force production (Geiger et al., 2000) by decreasing the number of available myosin heads to form strongly-bound XBs. However, more recent studies in the same animal models, as well as patients with cancer, have found no reduction in myosin protein content (Cosper and Leinwand, 2012; Toth et al., 2013). In fact, one study showed that prior findings of a select loss of myosin were likely a methodological artifact (Cosper and Leinwand, 2012). Notably, MHC isoform distribution also remains unchanged with cancer (Toth et al., 2013). Thus, from a quantitative perspective, cancer reduces overall muscle function primarily through its effect to decrease the overall mass of myofilament protein in muscle via simple atrophy, seemingly without selective loss of the major myofilament proteins myosin and actin. This gross loss of contractile tissue manifests functionally as muscle weakness, which undoubtedly contributes to physical disability in cancer patients.

### Muscle quality

Because of the prevalence of muscle atrophy in the cancer population, it is difficult to discern whether impaired muscle performance is related to a fundamental defect in contractility, or relates instead to the loss of the contractile components (i.e., myofilaments). In humans, to our knowledge, only three studies have simultaneously evaluated skeletal muscle contractile function and muscle size, with one study showing that muscle weakness was completely explained by atrophy (Weber et al., 2009), whereas the others suggested that there was a reduction in intrinsic contractility, or force production per unit muscle size (Stephens et al., 2012; Toth et al., 2013). Further reinforcing the notion of intrinsic contractile dysfunction, one recent study (Gallagher et al., 2012) reported that surgical removal of the tumor in colorectal cancer patients increased knee extensor isometric torque (~20%; personal communication with the authors) after a mean follow-up of 8 months despite reductions in body weight (and presumably muscle mass). Thus, from whole muscle measurements, the balance of evidence suggests that cancer is associated with some degree of intrinsic skeletal muscle contractile dysfunction.

Evidence to support intrinsic contractile dysfunction in cancer patients comes from studies at the single fiber level (Toth et al., 2013). We recently found evidence for contractile dysfunction in both MHC I and IIA fibers. In MHC IIA fibers, a reduction (~15%) in isometric tension was observed in cancer patients compared to fibers from healthy controls, which has been corroborated by a recent study from another laboratory (Taskin et al., 2014). These results provide strong evidence for intrinsic contractile dysfunction in cancer. Molecular level functional assessments suggested a potential mechanism for decreased isometric tension; namely, that the number of strongly-attached myosin-actin XBs was reduced in cancer patients, as single fiber force production is proportional to the number of strongly-bound XBs during Ca^2+^ activation. In addition to reduced tension in MHC IIA fibers, myosin-actin cross-bride kinetics were reduced in MHC I fibers (Toth et al., 2013). Specifically, myosin attachment time was increased in MHC I fibers from cancer patients compared to controls. From a functional standpoint, we would predict that this reduction in XB kinetics could decrease single fiber power output, as an increase in myosin attachment time would decrease single fiber contractile velocity (Piazzesi et al., 2007). Thus, this molecular level alteration may have functional significance to cellular and tissue level function. In support of this notion, we found that slower myosin rate of force production, another marker of reduced myosin-actin XB kinetics, were related to reduced knee extensor torque (Toth et al., 2013).

The mechanisms underlying intrinsic skeletal muscle contractile dysfunction with cancer are unclear. Data from whole muscle studies, in which knee extensor strength was measured before and after surgical removal of tumors indicates that alterations in function occur independent of changes in overall muscle size [i.e., improved function occurred in the setting of continued weight, and presumably muscle, loss (Stephens et al., 2012)]. In other words, intrinsic contractile dysfunction is not dependent upon or related to the atrophy process. One potential mediator that could explain contractile dysfunction is oxidative stress, as cancer is associated with increased oxidative stress in skeletal muscle (Barreiro et al., 2005; Ramamoorthy et al., 2009; Marin-Corral et al., 2010). Increased oxidant activity could promote contractile dysfunction via post-translational modification of myofilament proteins (Reid and Moylan, 2011). Indeed, pre-clinical studies that have treated skinned muscle fibers with oxidants have shown that oxidative modification of myofilament proteins reduces tension, velocity, myosin ATPase/XB kinetics and strongly-bound XBs (Wilson et al., 1991; Galler et al., 1997; Perkins et al., 1997; Callahan et al., 2001; Heunks et al., 2001; Coirault et al., 2007), effects that resemble the myofilament functional phenotype we observe in cancer patients (Toth et al., 2013). In support of this mechanism, we recently found that treatment of human single MHC I muscle fibers with *N*-ethylmaleimide (NEM) slowed XB kinetics, as evidenced by increased myosin attachment time. In fact, by down-titrating the NEM, which modifies protein thiol groups, a common target of cellular oxidants, we showed that we could reduce myosin kinetics with no change in single fiber tension (Callahan et al., 2014b), a phenotype that bears remarkable resemblance to the adaptations we observed in cancer patients. Additionally, cancer patients exhibited profound mitochondrial rarefaction and remodeling, with the former being associated with reduced XB kinetics in MHC I fibers and tending to be related to reduced strongly-bound XBs in MHC IIA fibers (Toth et al., 2013). Taken together, these results suggest disruption in mitochondrial biology, and possibly increased oxidant activity, in the skeletal muscle of cancer patients may contribute to myofilament dysfunction in both fiber types. As oxidative stress has also been forwarded as a potential mediator of muscle atrophy in cancer patients (Buck and Chojkier, 1996; Di Marco et al., 2005), our results suggest increased oxidant activity as a potential common mediator of the complex muscle phenotype of muscle atrophy and contractile dysfunction in cancer.

## Chronic obstructive pulmonary disease (COPD)

COPD is a lung disorder characterized by progressive airflow obstruction from inflammation and remodeling of the airways, which often results from emphysema secondary to cigarette smoking. Like heart failure, exercise intolerance is the cardinal symptom, which, along with the obvious contribution of the primary lung pathology, is thought to be due, in part, to a combination of atrophy and reduced oxidative capacity of skeletal muscles (Maltais et al., 2014). In fact, some authors have suggested similar pathoetiology of peripheral skeletal muscle adaptations in the two conditions (Gosker et al., 2000). The majority of research on peripheral skeletal muscle adaptations in COPD patients has focused on oxidative adaptations and atrophy and, to our knowledge, no study has specifically characterized myofilament function. In contrast, a considerable amount of work has been done examining diaphragm muscle myofilament protein expression and function, including two excellent studies evaluating single fiber function (Ottenheijm et al., 2005; Stubbings et al., 2008). This focus is logical considering the importance of respiratory muscle function in COPD. However, respiratory and peripheral skeletal muscle undergo very different adaptations in response to COPD (Levine et al., 2013), owing to the different pathophysiological demands—increased airway resistance necessitating increased muscle work in respiratory muscles and decreased muscle use owing to reduced physical activity in peripheral skeletal muscles (Watz et al., in press). For the purposes of the current review, we have focused primarily on studies that have evaluated myofilament adaptations in peripheral skeletal muscles.

### Muscle quantity and structure

Muscle atrophy in patients with COPD has largely been inferred from cross-sectional studies comparing whole muscle size in patients to non-diseased controls, with findings of reduced muscle size in COPD patients that is exacerbated with more severe disease (Remels et al., 2013; Maltais et al., 2014). These results are corroborated at the cellular level, with several studies showing decreased single muscle fiber size in COPD patients (Whittom et al., 1998; Gosker et al., 2002; Fermoselle et al., 2012). Interestingly, a recent study showed a similar rate of decline in lean tissue mass (a proxy of muscle mass) in individuals with COPD vs. non-diseased controls (Van Den Borst et al., 2011), suggesting that some differences in muscle fiber size may relate to early adaptations to cigarette smoking and/or could be a manifestation of the population that goes on to develop COPD. Regardless of the etiology, a loss of muscle fiber size would have clear relevance to decreased functional capacity and contribute to disability.

Numerous studies have suggested that that there is a skeletal muscle fiber type shift in COPD toward a more fast-twitch phenotype, manifested as a reduced relative expression of MHC I vs. MHC II fiber types (Gosker et al., 2007), with accompanying shifts in myosin light chain isoforms (Satta et al., 1997). Some studies have failed to find such a fiber type shift in deltoid muscle (Gea et al., 2001), arguing that fiber type adaptations to COPD in the lower extremity musculature may reflect the effects of muscle disuse. Thus, similar to heart failure, as discussed above, this fiber type adaptation may simply be function of chronic disuse in COPD patients, rather than an effect of the disease process *per se*.

### Muscle quality

As mentioned above, there are few studies that have directly measured muscle fiber function in COPD patients and nearly all of these have evaluated diaphragm muscle (Levine et al., 2003; Ottenheijm et al., 2005; Stubbings et al., 2008). There has been one study that evaluated function of isolated vastus lateralis muscle fiber bundles in COPD patients and controls (Debigare et al., 2003), which found no differences in maximal isometric tetanic contraction or adaptation in the force-frequency response, albeit the latter tended to be shifted to the right in COPD patients. Importantly, this study utilized an excitable muscle preparation, meaning that the contractile response reflects the effects of COPD on both myofilaments and Ca^2+^ release dynamics. Thus, the altered force-frequency response could be explained by alterations in Ca^2+^ release/reuptake, myofilament Ca^2+^ sensitivity or a combination of these changes. Caution is urged in interpreting this study, however, as the viability of an excitable muscle fiber bundle preparation is questionable.

In diaphragm muscle fibers, early studies in two COPD patients showed reduced single fiber tension (Levine et al., 2003). Studies in a larger population (*n* = 8) similarly showed reduced single fiber tension in MHC I and IIA fibers from COPD patients compared to controls and this reduction was attributed primarily to a reduction in myosin protein content (Ottenheijm et al., 2005). Moreover, the rate constant for force redevelopment following a following a rapid length decrease, a proxy measure of cross-bridge kinetics, was slower in COPD patients. A more recent study has further corroborated slowed cross-bridge cycling, as the ATPase rate of MHC I and IIA fibers was lower in COPD patients (Stubbings et al., 2008), although this study did not observe altered single fiber tension. Moreover, Stubbings et al. found reduced maximal power production from MHC IIA fibers in COPD patients. Collectively, these decrements in respiratory muscle function could limit pulmonary function and, in turn, contribute to exercise intolerance, the hallmark symptom of COPD and a clear contributor to disability. How these adaptations in myofilament function in diaphragm fibers reflect peripheral skeletal muscle myofilament adaptations, however, is unclear. Although there are some myofilament adaptations with COPD that generalize to striated skeletal muscle [e.g., myofilament protein oxidation (Caron et al., 2009)] and could reasonably be expected to impact function, as discussed above in regards to cancer-related myofilament dysfunction (Callahan et al., 2014b), studies on skinned single fibers from peripheral muscles of COPD patients are needed to confirm involvement of the myofilaments in reduced peripheral muscle contractile function that is also believed to contribute to disability (Maltais et al., 2014).

## Disuse

The specific adaptations of muscle to acute or chronic disuse are particularly interesting to physiologists and clinicians due to: (1) the profound effects of inactivity on multiple aspects of muscle size, protein composition and function and (2) the relevance of disuse for skeletal muscle adaptations to numerous clinically-relevant conditions (e.g., acute hospitalization, surgical recovery, chronic disease, etc.). Because chronic physical inactivity is both a risk factor and consequence of aging- and disease-related changes in skeletal muscle, it is difficult to disentangle the relative influence of aging/disease vs. disuse. As the effects of disuse have been studied rather extensively in healthy, younger humans (Adams et al., 2003; Narici and De Boer, 2011), this review will focus on adaptations to muscle disuse in older adult humans, which has received increased attention recently. Before beginning this discussion, we need to define acute and chronic disuse. As there are no widely-accepted definitions for the length of either acute or chronic or any biological hallmark that serves as a temporal cut-point, we have decided to put these into the context of clinically-relevant events that may impose disuse upon older adults. Acute periods of disuse occur with the onset or exacerbation of disease or other clinical events (e.g., surgery) and their subsequent period of convalescence. These can last for a few days, but may persist for several months (e.g., 3–6 months), as in the case of recovery from surgical interventions (e.g., joint replacement, hip fracture, coronary artery bypass graft surgery), with the defining characteristic being that muscle use patterns eventually return to “normal,” pre-event levels. In contrast, chronic muscle disuse is marked by a failure to re-establish prior levels of muscle use, with accommodation at a lower absolute level of muscle use. Therefore, by definition, this type of disuse lasts for years and may never be fully remediated.

### Muscle quantity/structure

Muscle disuse is known to cause marked reductions in muscle CSA in healthy young individuals (Adams et al., 2003; Narici and De Boer, 2011) and recent studies suggest that bed rest-induced muscle disuse is similarly associated with lower extremity muscle atrophy (−6%) in older adults (Kortebein et al., 2007). More recent studies that imposed disuse using leg casting in older adult men for a shorter period of time (4 d) have shown more robust atrophy (~10% reduction in both MHC I and II fiber types) compared to baseline when single muscle fiber CSA was evaluated (Suetta et al., 2012). Interestingly, when casting is maintained for 14 d, the degree of atrophy did not increase dramatically (~13% reduction) and this differs from younger men, who showed a more marked reduction in single muscle fiber CSA (−20%) (Suetta et al., 2012). Thus, disuse-related atrophy may be attenuated in older adults over time. In general, this atrophy was evenly distributed between MHC I and II fibers in shorter-term studies (4–5 d), but was more pronounced in MHC II fibers over longer periods (14 d) (Suetta et al., 2012; Wall et al., 2014). These data suggest that, like their younger counterparts, older adults appear to have a relatively robust atrophy response to experimentally-induced, acute muscle disuse. Additionally, one study showed that myosin protein content of single muscle fibers was profoundly reduced (−44% in MHC I and −31% in MHC IIA fibers, albeit differences in MHC II fibers did not reach significance) in two older adult men who were studied 3 months following leg immobilization for clinical purposes (D'Antona et al., 2003), but corroboration of these findings or characterization of the anatomical phenotype of myosin loss is lacking. Despite changes in muscle size, short term immobilization did not alter myosin isoform distribution (Hvid et al., 2010), while longer immobilization specifically increased MHC IIX expression (D'Antona et al., 2003).

Few studies have explored the effects of chronic muscle disuse on skeletal muscle size and structure in humans, primarily because experimentally-imposed disuse for extended periods of time on humans of any age would be deemed unethical. However, one could argue that this type of muscle disuse is very relevant to the muscle adaptations that occur in disease states (e.g., heart failure, COPD, etc.), where activity levels are lower than their non-diseased counterparts (Toth et al., 1997; Watz et al., in press). To study chronic disuse, several laboratories have evaluated patients with osteoarthritis, where physical inactivity is chronically depressed due to joint pain. If patients are selected to be free of other confounding pathologies, these patients could be considered a model of the effects of chronic disuse on skeletal muscle. Studies have shown that these patients have lower (~9%) quadriceps CSA in their limb with osteoarthritis compared to their unaffected limb (Suetta et al., 2007), and a greater degree of atrophy compared to non-diseased controls (Callahan et al., 2014b). Moreover, at the cellular level, there was profound atrophy (−14 to 32% depending on fiber type) in muscle fibers. Here again, the extent of atrophy is greater when examined at the single fiber vs. the tissue level. To date, no study has reported the effects of either acute or chronic muscle disuse on myofilament ultrastructure or other sub-cellular components.

### Muscle quality

Skeletal muscle performance is impaired with disuse in younger healthy adults, reflected in reduced whole muscle force and power production (Narici and De Boer, 2011), and this is similarly observed in older healthy adults in response to short periods of bed rest (Kortebein et al., 2008). That these changes may be associated with adaptations in myofilament function is suggested by several recent studies showing that chemically-skinned, single muscle fiber contractile function is impaired in older adults with acute muscle disuse. Single fiber tension after 4 and 14 days of leg casting is reduced similarly in young and older men in both MHC I and IIA fibers (Hvid et al., 2011, 2013), although reductions in MHC I fibers did not reach significance in response to 14 d of disuse. There were differential responses in Ca^2+^ sensitivity in young and older adult men that showed fiber type differences, with reductions in older men in MHC I and in young men in MHC IIA fibers, suggesting the possibility for age-related differences in the adaptations in actin-associated regulatory proteins. These findings provide important contributions to our understanding of the effects of acute disuse and encourage further study of how this combination of physiologic responses manifest to change *in vivo* voluntary torque production and mobility in at-risk populations, particularly as it pertains to acute hospitalization.

Building on these results, our recent studies suggest the functional effects of chronic disuse vary by sex (Callahan et al., 2014b). Although we similarly found reductions in single muscle fiber tension in older adults compared to active controls, these differences were only apparent in MHC IIA fibers and dissipated when fibers were evaluated at temperatures closer to *in vivo* conditions (25°C), suggesting that the effects of more chronic disuse on myofilament force production per unit fiber CSA is limited. In contrast, we found differences in contractile velocity; more specifically, that contractile velocity was reduced in females, but actually increased in males with chronic disuse. Perhaps most importantly, these differences in contractile velocity translated to reduced single fiber power output in women relative to men. Greater contractile velocity (and, in turn, power output) in men was accompanied by faster cross-bridge kinetics, as indicated by a reduced myosin attachment time and greater rate of myosin force production, suggesting that adaptations in molecular contractile function translate to the cellular level.

Do these adaptations in myofilament function at the molecular and cellular level contribute to the whole muscle phenotype of reduced power output in the lower extremity musculature? Extrapolation from the myofilament to the whole muscle level is difficult, as there are changes in numerous other physiological systems with disuse that regulate whole muscle torque (Narici and De Boer, 2011). Recent studies suggest that acute disuse has more prominent effects in older adults on dynamic muscle contractions with higher power output when compared to lower power output and isometric contractions (Hvid et al., 2014), which is in keeping with results in aging studies showing greater impairments in dynamic muscle contractile function and, in particular, higher velocity dynamic contractions (Lanza et al., 2003; Callahan and Kent-Braun, 2011; Miller et al., 2013). This may be explained by more pronounced slowing of cross-bridge kinetics in older adults, an adaptation that is larger in older adult women (Miller et al., 2013). With more chronic disuse, however, there may be compensatory adaptations to maintain myofilament contractile function specifically in men (Callahan et al., 2014b), with women lacking such adaptations. These myofilament adpatations with disuse may serve to diminish whole muscle deteroration, as recent longitudinal studies have shown that reductions in whole muscle function over time are accompanied by improvements in single muscle fiber myofilament force, velocity and power output (Reid et al., 2014). A failure to undergo such myofilament adaptations may predispose women to a more rapid development of disability. Thus, rather than a straight forward relationship of reduced myofilament function to decreased whole muscle function and, in turn, disability, variation in compensatory *increases* in myofilament function with longer-term disuse may explain a greater disposition toward lower extremity muscle function and disability in some individuals. This scenario provides potential cellular and molecular level mechanisms underlying higher disability rates in older women (Jette and Branch, 1981) and suggests that it may be important to consider the sex-specific response to disuse in men and women to completely characterize the functional phenotype. Notably, all studies examining the effect of acute disuse have been conducted in men, which makes it difficult to discern whether sex-specific adaptations are a feature of both acute and chronic disuse. Nonetheless, these findings show that myofilament adaptations may play an important role in the whole muscle and disability phenotypes that accompany muscle disuse.

## Cellular/molecular effects on whole muscle function

The contribution of myofilament adaptations to whole muscle contractile dysfunction and, in turn, physical disability with aging, disease, and disuse is difficult to discern because of the myriad of physiological systems that regulate whole muscle performance. Despite this multitude of confounding, higher level regulatory systems, several studies have shown relationships between myofilament properties and whole muscle performance. In older adults, a positive, but non-linear, relationship was found between single fiber and whole muscle tension (Frontera et al., 2000), indicating that the age-related decrease in single fiber performance may be partially responsible for decrements in whole muscle function. In our recent work examining young and older populations matched for physical activity level, although isokinetic knee torque was reduced with age, we found no differences in whole muscle isometric performance or relationships between isometric performance and single fiber functional parameters. However, slower XB kinetics and increased single fiber isometric tension in MHC IIA fibers predicted lower whole muscle isokinetic power with aging (Miller et al., 2013). Thus, age-related myofilament adaptations were correlated with the primary decrement in whole muscle performance with age. Similarly in cancer patients, reduced whole muscle isometric torque was related to slower XB kinetics (Toth et al., 2013). Both the cancer and aging populations also showed a relationship between slower XB kinetics and mitochondrial fractional area or size (Toth et al., 2013; Callahan et al., 2014a), suggesting the intriguing hypothesis that maladaptations in cellular energy homeostasis may contribute to myofilament dysfunction via post-translational oxidative modifications. Finally, in knee osteoarthritis patients, variation in XB kinetics in MHC IIA fibers scaled to the cellular level to yield sex-specific differences in single fiber power output (Callahan et al., 2014b). These molecular and cellular level differences were reflected in a similar pattern of differences in isokinetic torque at the whole muscle (i.e., lower torque in female knee osteoarthritis patients), albeit these tissue-level differences did not reach statistical significance, possibly due to our small sample size and/or sex-specific effects of pain-induced neural inhibition associated with knee osteoarthritis. Although preliminary, these studies collectively demonstrate that alterations at the molecular and cellular levels generally scale to the tissue level, suggesting that adaptations in myofilament protein expression and function may partially explain reductions in muscle function and lead to greater rates of physical functional disability with aging and disease.

## Summary

Aging, disease, and disuse alter single fiber and myofilament structure and function, although the changes vary depending upon the specific disease/physiological condition and sex. Structurally, the most significant changes were the loss of myosin content in heart failure patients and the general loss of CSA in a number of conditions (Table 1). Functionally, the parameter that was consistently changed with these conditions was XB kinetics (Table 2). Although XB kinetics tend to decrease in most instances, reductions are dependent upon sex and fiber type, with chronic muscle disuse in MHC IIA fibers in older men being the exception to the rule. Several studies have found relationships between cellular and molecular level myofilament function and whole muscle function (Frontera et al., 2000; Miller et al., 2013; Toth et al., 2013), underscoring their potential importance. However, the effects of each condition on function at the molecular, cellular and whole muscle levels become less clear when examined across multiple studies within each condition. This is most clearly evident in variation in myofilament function with aging, where data suggest that single fiber tension and velocity increase, remain unchanged, and decrease with age. We posit that such variation is due to the modifying effects of confounding factors, such as the habitual activity level and sex distribution of the populations studied, which have not commonly been accounted for in experimental designs or statistical analyses. While integrated myofilament function has been evaluated in the context of single muscle fiber function, sometimes extending to the cross-bridge level, few studies have interrogated the specific changes in myofilament protein, expression and/or post-translational modification might be accounting for these adaptation. Overall, these findings indicate that a more thorough understanding of the myofilament adaptations to aging, disease, and disuse in both sexes would assist with the development of preventative and rehabilitative interventions to improve muscle function and, in turn, decrease functional disability in older men and women.

**Table 1:** **Summary of structural changes in single fiber and myofilament structure with aging, disease, and disuse**.

**Structure**	**Aging**	**Heart failure**	**Cancer**	**Disuse**
Ultrastructure	↔	↔	↔	n.a.
MHC content	↔	↓	↔	↔
MHC isoform phenotype	↔	↑ ↔	↔	↑ ↔
Cross-sectional area	↔ ↓	↔ ↓	↔ ↓	↔ ↓

↑, increase/faster; ↔, no change; ↓, decrease/slower; n.a., not available.

Ultrastructure includes thick filament length, thick-to-thin filament ratio, and myofibrillar volume fraction; MHC, myosin heavy chain.

**Table 2:** **Summary of functional changes in single fiber and myofilament structure with aging, disease, and disuse**.

**Function**	**Aging**	**Heart failure**	**Cancer**	**Disuse**
**MOLECULAR LEVEL**				
Cross-bridge kinetics	↓ ♀ > ♂	↓	↓ MHC I	↓ MHC I of ♀
			↔ MHC IIA	↑ MHC IIA of ♂
**SINGLE FIBER LEVEL**				
Isometric tension	↑ ↔ ↓	↔ ↓	↔ ↓	↔ ↓
Contractile velocity	↑ ↔ ↓	↓^*^	↓^*^	↔ MHC I
				↑ MHC IIA of ♂
				↓ MHC IIA of ♀

↑, increase/faster; ↔, no change, ↓, decrease/slower, ♂, male, ♀, female, >, greater than;

* predicted from XB kinetic differences.

### Conflict of interest statement

The authors declare that the research was conducted in the absence of any commercial or financial relationships that could be construed as a potential conflict of interest.

## References

[B1] Acharyya S. Ladner K. J. Nelsen L. L. Damrauer J. Reiser P. J. Swoap S. (2004). Cancer cachexia is regulated by selective targeting of skeletal muscle gene products. J. Clin. Invest. 114, 370–378 10.1172/JCI20174 15286803 PMC484974

[B2] Adams G. R. Caiozzo V. J. Baldwin K. M. (2003). Skeletal muscle unweighting: spaceflight and ground-based models. J. Appl. Physiol. (1985) 95, 2185–2201 10.1152/japplphysiol.00346.2003. 14600160

[B3] Barreiro E. De La Puente B. Busquets S. Lopez-Soriano F. J. Gea J. Argiles J. M. (2005). Both oxidative and nitrosative stress are associated with muscle wasting in tumour-bearing rats. FEBS Lett. 579, 1646–1652 10.1016/j.febslet.2005.02.017 15757655

[B4] Blaauw B. Canato M. Agatea L. Toniolo L. Mammucari C. Masiero E. (2009). Inducible activation of Akt increases skeletal muscle mass and force without satellite cell activation. FASEB J. 23, 3896–3905 10.1096/fj.09-131870 19661286

[B5] Bottinelli R. (2001). Functional heterogeneity of mammalian single muscle fibres: do myosin isoforms tell the whole story? Pflugers Arch. 443, 6–17 10.1007/s004240100700 11692261

[B6] Brenner B. (1988). Effect of Ca^2+^ on cross-bridge turnover kinetics in skinned single rabbit psoas fibers: implications for regulation of muscle contraction. Proc. Natl. Acad. Sci. U.S.A. 85, 3265–3269 10.1073/pnas.85.9.3265 2966401 PMC280185

[B7] Brenner B. Hahn N. Hanke E. Matinmehr F. Scholz T. Steffen W. (2012). Mechanical and kinetic properties of beta-cardiac/slow skeletal muscle myosin. J. Muscle Res. Cell Motil. 33, 403–417 10.1007/s10974-012-9315-8 22847802

[B8] Buck M. Chojkier M. (1996). Muscle wasting and dedifferentiation induced by oxidative stress in a murine model of cachexia is prevented by inhibitors of nitric oxide synthesis and antioxidants. EMBO J. 15, 1753–1765 8617220 PMC450091

[B9] Callahan D. M. Bedrin N. G. Subramanian M. Berking J. Ades P. A. Toth M. J. (2014a). Age-related structural alterations in human skeletal muscle fibers and mitochondria are sex specific: relationship to single-fiber function. J. Appl. Physiol. (1985) 116, 1582–1592 10.1152/japplphysiol.01362.2013 24790014 PMC4064376

[B10] Callahan D. M. Kent-Braun J. A. (2011). Effect of old age on human skeletal muscle force-velocity and fatigue properties. J. Appl. Physiol. (1985) 111, 1345–1352 10.1152/japplphysiol.00367.2011 21868683 PMC3220307

[B11] Callahan D. M. Miller M. S. Sweeney A. P. Tourville T. W. Slauterbeck J. R. Savage P. D. (2014b). Muscle disuse alters skeletal muscle contractile function at the molecular and cellular levels in older adult humans in a sex-specific manner. J. Physiol. 10.1113/jphysiol.2014.279034 PMC4287744 25038243

[B12] Callahan L. A. She Z. W. Nosek T. M. (2001). Superoxide, hydroxyl radical, and hydrogen peroxide effects on single-diaphragm fiber contractile apparatus. J. Appl. Physiol. (1985) 90, 45–54 11133892 10.1152/jappl.2001.90.1.45

[B13] Calvani R. Joseph A. M. Adhihetty P. J. Miccheli A. Bossola M. Leeuwenburgh C. (2013). Mitochondrial pathways in sarcopenia of aging and disuse muscle atrophy. Biol. Chem. 394, 393–414 10.1515/hsz-2012-0247 23154422 PMC3976204

[B14] Canepari M. Rossi R. Pellegrino M. A. Orrell R. W. Cobbold M. Harridge S. (2005). Effects of resistance training on myosin function studied by the *in vitro* motility assay in young and older men. J. Appl. Physiol. (1985) 98, 2390–2395 10.1152/japplphysiol.01103.2004 15677736

[B15] Capitanio M. Canepari M. Cacciafesta P. Lombardi V. Cicchi R. Maffei M. (2006). Two independent mechanical events in the interaction cycle of skeletal muscle myosin with actin. Proc. Natl. Acad. Sci. U.S.A. 103, 87–92 10.1073/pnas.0506830102 16371472 PMC1324983

[B16] Caron M. A. Debigare R. Dekhuijzen P. N. Maltais F. (2009). Comparative assessment of the quadriceps and the diaphragm in patients with COPD. J. Appl. Physiol. (1985) 107, 952–961 10.1152/japplphysiol.00194.2009 19359618

[B17] Cella D. Davis K. Breitbart W. Curt G. Fatigue C. (2001). Cancer-related fatigue: prevalence of proposed diagnostic criteria in a United States sample of cancer survivors. J. Clin. Oncol. 19, 3385–3391 11454886 10.1200/JCO.2001.19.14.3385

[B18] Coirault C. Guellich A. Barbry T. Samuel J. L. Riou B. Lecarpentier Y. (2007). Oxidative stress of myosin contributes to skeletal muscle dysfunction in rats with chronic heart failure. Am. J. Physiol. Heart Circ. Physiol. 292, H1009–H1017 10.1152/ajpheart.00438.2006 17040975

[B19] Cosper P. F. Leinwand L. A. (2012). Myosin heavy chain is not selectively decreased in murine cancer cachexia. Int. J. Cancer 130, 2722–2727 10.1002/ijc.26298 21796617 PMC3267878

[B20] D'Antona G. Lanfranconi F. Pellegrino M. A. Brocca L. Adami R. Rossi R. (2006). Skeletal muscle hypertrophy and structure and function of skeletal muscle fibres in male body builders. J. Physiol. 570, 611–627 10.1113/jphysiol.2005.101642 16339176 PMC1479884

[B21] D'Antona G. Pellegrino M. A. Adami R. Rossi R. Carlizzi C. N. Canepari M. (2003). The effect of ageing and immobilization on structure and function of human skeletal muscle fibres. J. Physiol. 552, 499–511 10.1113/jphysiol.2003.046276 14561832 PMC2343394

[B22] D'Antona G. Pellegrino M. A. Carlizzi C. N. Bottinelli R. (2007). Deterioration of contractile properties of muscle fibres in elderly subjects is modulated by the level of physical activity. Eur. J. Appl. Physiol. 100, 603–611 10.1007/s00421-007-0402-2 17273882

[B23] Debigare R. Cote C. H. Hould F. S. Leblanc P. Maltais F. (2003). *In vitro* and *in vivo* contractile properties of the vastus lateralis muscle in males with COPD. Eur. Respir. J. 21, 273–278 10.1183/09031936.03.00036503 12608441

[B24] Delmonico M. J. Harris T. B. Visser M. Park S. W. Conroy M. B. Velasquez-Mieyer P. (2009). Longitudinal study of muscle strength, quality, and adipose tissue infiltration. Am. J. Clin. Nutr. 90, 1579–1585 10.3945/ajcn.2009.28047 19864405 PMC2777469

[B25] Di Marco S. Mazroui R. Dallaire P. Chittur S. Tenenbaum S. A. Radzioch D. (2005). NF-kappa B-mediated MyoD decay during muscle wasting requires nitric oxide synthase mRNA stabilization, HuR protein, and nitric oxide release. Mol. Cell. Biol. 25, 6533–6545 10.1128/MCB.25.15.6533-6545.2005 16024790 PMC1190341

[B26] Eley H. L. Skipworth R. J. Deans D. A. Fearon K. C. Tisdale M. J. (2008). Increased expression of phosphorylated forms of RNA-dependent protein kinase and eukaryotic initiation factor 2alpha may signal skeletal muscle atrophy in weight-losing cancer patients. Br. J. Cancer 98, 443–449 10.1038/sj.bjc.6604150 18087277 PMC2361431

[B27] Fearon K. C. Glass D. J. Guttridge D. C. (2012). Cancer cachexia: mediators, signaling, and metabolic pathways. Cell Metab. 16, 153–166 10.1016/j.cmet.2012.06.011 22795476

[B28] Fermoselle C. Rabinovich R. Ausin P. Puig-Vilanova E. Coronell C. Sanchez F. (2012). Does oxidative stress modulate limb muscle atrophy in severe COPD patients? Eur. Respir. J. 40, 851–862 10.1183/09031936.00137211 22408199

[B29] Franchi L. L. Murdoch A. Brown W. E. Mayne C. N. Elliott L. Salmons S. (1990). Subcellular localization of newly incorporated myosin in rabbit fast skeletal muscle undergoing stimulation-induced type transformation. J. Muscle Res. Cell Motil. 11, 227–239 10.1007/BF01843576 2401723

[B30] Frontera W. R. Hughes V. A. Krivickas L. S. Kim S. K. Foldvari M. Roubenoff R. (2003). Strength training in older women: early and late changes in whole muscle and single cells. Muscle Nerve 28, 601–608 10.1002/mus.10480 14571463

[B31] Frontera W. R. Reid K. F. Phillips E. M. Krivickas L. S. Hughes V. A. Roubenoff R. (2008). Muscle fiber size and function in elderly humans: a longitudinal study. J. Appl. Physiol. (1985) 105, 637–642 10.1152/japplphysiol.90332.2008 18556434 PMC2519941

[B32] Frontera W. R. Suh D. Krivickas L. S. Hughes V. A. Goldstein R. Roubenoff R. (2000). Skeletal muscle fiber quality in older men and women. Am. J. Physiol. Cell Physiol. 279, C611–C618 10942711 10.1152/ajpcell.2000.279.3.C611

[B33] Gallagher I. J. Stephens N. A. Macdonald A. J. Skipworth R. J. Husi H. Greig C. A. (2012). Suppression of skeletal muscle turnover in cancer cachexia: evidence from the transcriptome in sequential human muscle biopsies. Clin. Cancer Res. 18, 2817–2827 10.1158/1078-0432.CCR-11-2133 22452944

[B34] Galler S. Hilber K. Gobesberger A. (1997). Effects of nitric oxide on force-generating proteins of skeletal muscle. Pflugers Arch. 434, 242–245 10.1007/s004240050391 9178621

[B35] Gea J. G. Pasto M. Carmona M. A. Orozco-Levi M. Palomeque J. Broquetas J. (2001). Metabolic characteristics of the deltoid muscle in patients with chronic obstructive pulmonary disease. Eur. Respir. J. 17, 939–945 10.1183/09031936.01.17509390 11488330

[B36] Geiger P. C. Cody M. J. Macken R. L. Sieck G. C. (2000). Maximum specific force depends on myosin heavy chain content in rat diaphragm muscle fibers. J. Appl. Physiol. (1985) 89, 695–703 10926656 10.1152/jappl.2000.89.2.695

[B37] Gelfi C. Vigano A. Ripamonti M. Pontoglio A. Begum S. Pellegrino M. A. (2006). The human muscle proteome in aging. J. Proteome Res. 5, 1344–1353 10.1021/pr050414x 16739986

[B38] Gosker H. R. Engelen M. P. Van Mameren H. Van Dijk P. J. Van Der Vusse G. J. Wouters E. F. (2002). Muscle fiber type IIX atrophy is involved in the loss of fat-free mass in chronic obstructive pulmonary disease. Am. J. Clin. Nutr. 76, 113–119 12081824 10.1093/ajcn/76.1.113

[B39] Gosker H. R. Wouters E. F. Van Der Vusse G. J. Schols A. M. (2000). Skeletal muscle dysfunction in chronic obstructive pulmonary disease and chronic heart failure: underlying mechanisms and therapy perspectives. Am. J. Clin. Nutr. 71, 1033–1047 10799364 10.1093/ajcn/71.5.1033

[B40] Gosker H. R. Zeegers M. P. Wouters E. F. Schols A. M. (2007). Muscle fibre type shifting in the vastus lateralis of patients with COPD is associated with disease severity: a systematic review and meta-analysis. Thorax 62, 944–949 10.1136/thx.2007.078980 17526675 PMC2117111

[B41] Gouzi F. Maury J. Molinari N. Pomies P. Mercier J. Prefaut C. (2013). Reference values for vastus lateralis fiber size and type in healthy subjects over 40 years old: a systematic review and metaanalysis. J. Appl. Physiol. (1985) 115, 346–354 10.1152/japplphysiol.01352.2012 23558383

[B42] Guilford W. H. Dupuis D. E. Kennedy G. Wu J. Patlak J. B. Warshaw D. M. (1997). Smooth muscle and skeletal muscle myosins produce similar unitary forces and displacements in the laser trap. Biophys. J. 72, 1006–1021 10.1016/S0006-3495(97)78753-8 9138552 PMC1184489

[B43] Guralnik J. M. Ferrucci L. Simonsick E. M. Salive M. E. Wallace R. B. (1995). Lower-extremity function in persons over the age of 70 years as a predictor of subsequent disability. N. Engl. J. Med. 332, 556–561 10.1056/NEJM199503023320902 7838189 PMC9828188

[B44] Harber M. P. Konopka A. R. Douglass M. D. Minchev K. Kaminsky L. A. Trappe T. A. (2009). Aerobic exercise training improves whole muscle and single myofiber size and function in older women. Am. J. Physiol. Regul. Integr. Comp. Physiol. 297, R1452–R1459 10.1152/ajpregu.00354.2009 19692660 PMC3774188

[B45] Harridge S. D. Bottinelli R. Canepari M. Pellegrino M. A. Reggiani C. Esbjornsson M. (1996). Whole-muscle and single-fibre contractile properties and myosin heavy chain isoforms in humans. Pflugers Arch. 432, 913–920 10.1007/s004240050215 8772143

[B46] Harrington D. Anker S. D. Chua T. P. Webb-Peploe K. M. Ponikowski P. P. Poole-Wilson P. A. (1997). Skeletal muscle function and its relation to exercise tolerance in chronic heart failure. J. Am. Coll. Cardiol. 30, 1758–1764 10.1016/S0735-1097(97)00381-1 9385904

[B47] Heunks L. M. Cody M. J. Geiger P. C. Dekhuijzen P. N. Sieck G. C. (2001). Nitric oxide impairs Ca^2+^ activation and slows cross-bridge cycling kinetics in skeletal muscle. J. Appl. Physiol. (1985) 91, 2233–2239 11641366 10.1152/jappl.2001.91.5.2233

[B48] Hooft A. M. Maki E. J. Cox K. K. Baker J. E. (2007). An accelerated state of myosin-based actin motility. Biochemistry 46, 3513–3520 10.1021/bi0614840 17302393

[B49] Hook P. Sriramoju V. Larsson L. (2001). Effects of aging on actin sliding speed on myosin from single skeletal muscle cells of mice, rats, and humans. Am. J. Physiol. Cell Physiol. 280, C782–C788 11245594 10.1152/ajpcell.2001.280.4.C782

[B50] Huxley A. F. (1957). Muscle structure and theories of contraction. Prog. Biophys. Biophys. Chem. 7, 255–318 13485191

[B51] Hvid L. Aagaard P. Justesen L. Bayer M. L. Andersen J. L. Ortenblad N. (2010). Effects of aging on muscle mechanical function and muscle fiber morphology during short-term immobilization and subsequent retraining. J. Appl. Physiol. (1985) 109, 1628–1634 10.1152/japplphysiol.00637.2010 20864557

[B52] Hvid L. G. Ortenblad N. Aagaard P. Kjaer M. Suetta C. (2011). Effects of ageing on single muscle fibre contractile function following short-term immobilisation. J. Physiol. 589, 4745–4757 10.1113/jphysiol.2011.215434 21825028 PMC3213421

[B53] Hvid L. G. Suetta C. Aagaard P. Kjaer M. Frandsen U. Ortenblad N. (2013). Four days of muscle disuse impairs single fiber contractile function in young and old healthy men. Exp. Gerontol. 48, 154–161 10.1016/j.exger.2012.11.005 23220118

[B54] Hvid L. G. Suetta C. Nielsen J. H. Jensen M. M. Frandsen U. Ortenblad N. (2014). Aging impairs the recovery in mechanical muscle function following 4 days of disuse. Exp. Gerontol. 52, 1–8 10.1016/j.exger.2014.01.012 24447828

[B55] Janssen I. Heymsfield S. B. Ross R. (2002). Low relative skeletal muscle mass (sarcopenia) in older persons is associated with functional impairment and physical disability. J. Am. Geriatr. Soc. 50, 889–896 10.1046/j.1532-5415.2002.50216.x 12028177

[B56] Jette A. M. Branch L. G. (1981). The Framingham disability study: II. Physical disability among the aging. Am. J. Public Health 71, 1211–1216 10.2105/AJPH.71.11.1211 7294262 PMC1619934

[B57] Jubrias S. A. Odderson I. R. Esselman P. C. Conley K. E. (1997). Decline in isokinetic force with age: muscle cross-sectional area and specific force. Pflugers Arch. 434, 246–253 10.1007/s004240050392 9178622

[B58] Korhonen M. T. Cristea A. Alen M. Hakkinen K. Sipila S. Mero A. (2006). Aging, muscle fiber type, and contractile function in sprint-trained athletes. J. Appl. Physiol. (1985) 101, 906–917 10.1152/japplphysiol.00299.2006 16690791

[B59] Kortebein P. Ferrando A. Lombeida J. Wolfe R. Evans W. J. (2007). Effect of 10 days of bed rest on skeletal muscle in healthy older adults. JAMA 297, 1772–1774 10.1001/jama.297.16.1772-b 17456818

[B60] Kortebein P. Symons T. B. Ferrando A. Paddon-Jones D. Ronsen O. Protas E. (2008). Functional impact of 10 days of bed rest in healthy older adults. J. Gerontol. A Biol. Sci. Med. Sci. 63, 1076–1081 10.1093/gerona/63.10.1076 18948558

[B61] Krivickas L. S. Dorer D. J. Ochala J. Frontera W. R. (2011). Relationship between force and size in human single muscle fibres. Exp. Physiol. 96, 539–547 10.1113/expphysiol.2010.055269 21317219

[B62] Krivickas L. S. Fielding R. A. Murray A. Callahan D. Johansson A. Dorer D. J. (2006). Sex differences in single muscle fiber power in older adults. Med. Sci. Sports Exerc. 38, 57–63 10.1249/01.mss.0000180357.58329.b1 16394954

[B63] Krivickas L. S. Suh D. Wilkins J. Hughes V. A. Roubenoff R. Frontera W. R. (2001). Age- and gender-related differences in maximum shortening velocity of skeletal muscle fibers. Am. J. Phys. Med. Rehabil. 80, 447–455; quiz: 456–447. 10.1097/00002060-200106000-00012 11399006

[B64] Lanza I. R. Towse T. F. Caldwell G. E. Wigmore D. M. Kent-Braun J. A. (2003). Effects of age on human muscle torque, velocity, and power in two muscle groups. J. Appl. Physiol. (1985) 95, 2361–2369 10.1152/japplphysiol.00724.2002 12923120

[B65] Larsson L. Li X. Frontera W. R. (1997). Effects of aging on shortening velocity and myosin isoform composition in single human skeletal muscle cells. Am. J. Physiol. 272, C638–C649 9124308 10.1152/ajpcell.1997.272.2.C638

[B66] Levine S. Bashir M. H. Clanton T. L. Powers S. K. Singhal S. (2013). COPD elicits remodeling of the diaphragm and vastus lateralis muscles in humans. J. Appl. Physiol. (1985) 114, 1235–1245 10.1152/japplphysiol.01121.2012 23264538 PMC3656432

[B67] Levine S. Nguyen T. Kaiser L. R. Rubinstein N. A. Maislin G. Gregory C. (2003). Human diaphragm remodeling associated with chronic obstructive pulmonary disease: clinical implications. Am. J. Respir. Crit. Care Med. 168, 706–713 10.1164/rccm.200209-1070OC 12857719

[B68] Lexell J. (1995). Human aging, muscle mass, and fiber type composition. J. Gerontol. A Biol. Sci. Med. Sci. 50 Spec No: 11–16. 7493202 10.1093/gerona/50a.special_issue.11

[B69] Lexell J. Downham D. Y. (1991). The occurrence of fibre-type grouping in healthy human muscle: a quantitative study of cross-sections of whole vastus lateralis from men between 15 and 83 years. Acta Neuropathol. 81, 377–381 10.1007/BF00293457 2028741

[B70] Lexell J. Taylor C. C. Sjostrom M. (1988). What is the cause of the ageing atrophy? Total number, size and proportion of different fiber types studied in whole vastus lateralis muscle from 15- to 83-year-old men. J. Neurol. Sci. 84, 275–294 10.1016/0022-510X(88)90132-3 3379447

[B71] Linari M. Bottinelli R. Pellegrino M. A. Reconditi M. Reggiani C. Lombardi V. (2004). The mechanism of the force response to stretch in human skinned muscle fibres with different myosin isoforms. J. Physiol. 554, 335–352 10.1113/jphysiol.2003.051748 14555725 PMC1664769

[B72] Lindle R. S. Metter E. J. Lynch N. A. Fleg J. L. Fozard J. L. Tobin J. (1997). Age and gender comparisons of muscle strength in 654 women and men aged 20-93 yr. J. Appl. Physiol. (1985) 83, 1581–1587 9375323 10.1152/jappl.1997.83.5.1581

[B73] Lynch N. A. Metter E. J. Lindle R. S. Fozard J. L. Tobin J. D. Roy T. A. (1999). Muscle quality. I. Age-associated differences between arm and leg muscle groups. J. Appl. Physiol. (1985) 86, 188–194 9887130 10.1152/jappl.1999.86.1.188

[B74] Maltais F. Decramer M. Casaburi R. Barreiro E. Burelle Y. Debigare R. (2014). An official American Thoracic Society/European Respiratory Society statement: update on limb muscle dysfunction in chronic obstructive pulmonary disease. Am. J. Respir. Crit. Care Med. 189, e15–e62 10.1164/rccm.201402-0373ST 24787074 PMC4098112

[B75] Mancini D. M. Coyle E. Coggan A. Beltz J. Ferraro N. Montain S. (1989). Contribution of intrinsic skeletal muscle changes to 31P NMR skeletal muscle metabolic abnormalities in patients with chronic heart failure. Circulation 80, 1338–1346 10.1161/01.CIR.80.5.1338 2805270

[B76] Marin-Corral J. Fontes C. C. Pascual-Guardia S. Sanchez F. Olivan M. Argiles J. M. (2010). Redox balance and carbonylated proteins in limb and heart muscles of cachectic rats. Antioxid. Redox Signal. 12, 365–380 10.1089/ars.2009.2818 19737087

[B77] Massie B. M. Simonini A. Sahgal P. Wells L. Dudley G. A. (1996). Relation of systemic and local muscle exercise capacity to skeletal muscle characteristics in men with congestive heart failure. J. Am. Coll. Cardiol. 27, 140–145 10.1016/0735-1097(95)00416-5 8522687

[B78] Mettauer B. Zoll J. Sanchez H. Lampert E. Ribera F. Veksler V. (2001). Oxidative capacity of skeletal muscle in heart failure patients versus sedentary or active control subjects. J. Am. Coll. Cardiol. 38, 947–954 10.1016/S0735-1097(01)01460-7 11583863

[B79] Miller M. S. Bedrin N. G. Callahan D. M. Previs M. J. Jennings M. E. 2nd. Ades P. A. (2013). Age-related slowing of myosin actin cross-bridge kinetics is sex specific and predicts decrements in whole skeletal muscle performance in humans. J. Appl. Physiol. (1985) 115, 1004–1014 10.1152/japplphysiol.00563.2013 23887900 PMC3798822

[B80] Miller M. S. Toth M. J. (2013). Myofilament protein alterations promote physical disability in aging and disease. Exerc. Sport Sci. Rev. 41, 93–99 10.1097/JES.0b013e31828bbcd8 23392279 PMC4171103

[B81] Miller M. S. Vanburen P. Lewinter M. M. Braddock J. M. Ades P. A. Maughan D. W. (2010). Chronic heart failure decreases cross-bridge kinetics in single skeletal muscle fibres from humans. J. Physiol. 588, 4039–4053 10.1113/jphysiol.2010.191957 20724360 PMC3000591

[B82] Miller M. S. Vanburen P. Lewinter M. M. Lecker S. H. Selby D. E. Palmer B. M. (2009). Mechanisms underlying skeletal muscle weakness in human heart failure: alterations in single fiber myosin protein content and function. Circ. Heart Fail. 2, 700–706 10.1161/CIRCHEARTFAILURE.109.876433 19919996 PMC2782533

[B83] Morse C. I. Thom J. M. Reeves N. D. Birch K. M. Narici M. V. (2005). *In vivo* physiological cross-sectional area and specific force are reduced in the gastrocnemius of elderly men. J. Appl. Physiol. (1985) 99, 1050–1055 10.1152/japplphysiol.01186.2004 15905324

[B84] Narici M. V. De Boer M. D. (2011). Disuse of the musculo-skeletal system in space and on earth. Eur. J. Appl. Physiol. 111, 403–420 10.1007/s00421-010-1556-x 20617334

[B85] Ochala J. Dorer D. J. Frontera W. R. Krivickas L. S. (2006). Single skeletal muscle fiber behavior after a quick stretch in young and older men: a possible explanation of the relative preservation of eccentric force in old age. Pflugers Arch. 452, 464–470 10.1007/s00424-006-0065-6 16622703

[B86] Ochala J. Frontera W. R. Dorer D. J. Van Hoecke J. Krivickas L. S. (2007). Single skeletal muscle fiber elastic and contractile characteristics in young and older men. J. Gerontol. A Biol. Sci. Med. Sci. 62, 375–381 10.1093/gerona/62.4.375 17452730

[B87] Okada Y. Toth M. J. Vanburen P. (2008). Skeletal muscle contractile protein function is preserved in human heart failure. J. Appl. Physiol. (1985) 104, 952–957 10.1152/japplphysiol.01072.2007 18202167 PMC2735408

[B88] Ottenheijm C. A. Heunks L. M. Sieck G. C. Zhan W. Z. Jansen S. M. Degens H. (2005). Diaphragm dysfunction in chronic obstructive pulmonary disease. Am. J. Respir. Crit. Care Med. 172, 200–205 10.1164/rccm.200502-262OC 15849324 PMC2718467

[B89] Palmer B. M. Suzuki T. Wang Y. Barnes W. D. Miller M. S. Maughan D. W. (2007). Two-state model of acto-myosin attachment-detachment predicts C-process of sinusoidal analysis. Biophys. J. 93, 760–769 10.1529/biophysj.106.101626 17496022 PMC1913148

[B90] Palmiter K. A. Tyska M. J. Dupuis D. E. Alpert N. R. Warshaw D. M. (1999). Kinetic differences at the single molecule level account for the functional diversity of rabbit cardiac myosin isoforms. J. Physiol. 519 pt 3, 669–678 10.1111/j.1469-7793.1999.0669n.x 10457082 PMC2269540

[B91] Pansarasa O. Rinaldi C. Parente V. Miotti D. Capodaglio P. Bottinelli R. (2009). Resistance training of long duration modulates force and unloaded shortening velocity of single muscle fibres of young women. J. Electromyogr. Kinesiol. 19, e290–e300 10.1016/j.jelekin.2008.07.007 18801662

[B92] Parente V. D'Antona G. Adami R. Miotti D. Capodaglio P. De Vito G. (2008). Long-term resistance training improves force and unloaded shortening velocity of single muscle fibres of elderly women. Eur. J. Appl. Physiol. 104, 885–893 10.1007/s00421-008-0845-0 18677504

[B93] Pellegrino M. A. Canepari M. Rossi R. D'Antona G. Reggiani C. Bottinelli R. (2003). Orthologous myosin isoforms and scaling of shortening velocity with body size in mouse, rat, rabbit and human muscles. J. Physiol. 546, 677–689 10.1113/jphysiol.2002.027375 12562996 PMC2342590

[B94] Perkins W. J. Han Y. S. Sieck G. C. (1997). Skeletal muscle force and actomyosin ATPase activity reduced by nitric oxide donor. J. Appl. Physiol. (1985) 83, 1326–1332 9338443 10.1152/jappl.1997.83.4.1326

[B95] Piazzesi G. Reconditi M. Linari M. Lucii L. Bianco P. Brunello E. (2007). Skeletal muscle performance determined by modulation of number of myosin motors rather than motor force or stroke size. Cell 131, 784–795 10.1016/j.cell.2007.09.045 18022371

[B96] Pinsky J. L. Jette A. M. Branch L. G. Kannel W. B. Feinleib M. (1990). The Framingham disability study: relationship of various coronary heart disease manifestations to disability in older persons living in the community. Am. J. Public Health 80, 1363–1367 10.2105/AJPH.80.11.1363 2240306 PMC1404890

[B97] Pittman J. G. Cohen P. (1964). The pathogenesis of cardiac cachexia. N. Engl. J. Med. 271, 403–409 10.1056/NEJM196408202710807 14164660

[B98] Raj I. S. Bird S. R. Shield A. J. (2010). Aging and the force-velocity relationship of muscles. Exp. Gerontol. 45, 81–90 10.1016/j.exger.2009.10.013 19883746

[B99] Ramamoorthy S. Donohue M. Buck M. (2009). Decreased Jun-D and myogenin expression in muscle wasting of human cachexia. Am. J. Physiol. Endocrinol. Metab. 297, E392–E401 10.1152/ajpendo.90529.2008 19470832 PMC2724118

[B100] Rehn T. A. Munkvik M. Lunde P. K. Sjaastad I. Sejersted O. M. (2012). Intrinsic skeletal muscle alterations in chronic heart failure patients: a disease-specific myopathy or a result of deconditioning? Heart Fail. Rev. 17, 421–436 10.1007/s10741-011-9289-4 21996779

[B101] Reid K. F. Doros G. Clark D. J. Patten C. Carabello R. J. Cloutier G. J. (2012). Muscle power failure in mobility-limited older adults: preserved single fiber function despite lower whole muscle size, quality and rate of neuromuscular activation. Eur. J. Appl. Physiol. 112, 2289–2301 10.1007/s00421-011-2200-0 22005960 PMC3394542

[B102] Reid K. F. Fielding R. A. (2012). Skeletal muscle power: a critical determinant of physical functioning in older adults. Exerc. Sport Sci. Rev. 40, 4–12 10.1097/JES.0b013e31823b5f13 22016147 PMC3245773

[B103] Reid K. F. Pasha E. Doros G. Clark D. J. Patten C. Phillips E. M. (2014). Longitudinal decline of lower extremity muscle power in healthy and mobility-limited older adults: influence of muscle mass, strength, composition, neuromuscular activation and single fiber contractile properties. Eur. J. Appl. Physiol. 114, 29–39 10.1007/s00421-013-2728-2 24122149 PMC3945182

[B104] Reid M. B. Moylan J. S. (2011). Beyond atrophy: redox mechanisms of muscle dysfunction in chronic inflammatory disease. J. Physiol. 589, 2171–2179 10.1113/jphysiol.2010.203356 21320886 PMC3098696

[B105] Reiken S. Lacampagne A. Zhou H. Kherani A. Lehnart S. E. Ward C. (2003). PKA phosphorylation activates the calcium release channel (ryanodine receptor) in skeletal muscle: defective regulation in heart failure. J. Cell Biol. 160, 919–928 10.1083/jcb.200211012 12629052 PMC2173774

[B106] Reiser P. J. Kasper C. E. Moss R. L. (1987). Myosin subunits and contractile properties of single fibers from hypokinetic rat muscles. J. Appl. Physiol. (1985) 63, 2293–2300 2963800 10.1152/jappl.1987.63.6.2293

[B107] Remels A. H. Gosker H. R. Langen R. C. Schols A. M. (2013). The mechanisms of cachexia underlying muscle dysfunction in COPD. J. Appl. Physiol. (1985) 114, 1253–1262 10.1152/japplphysiol.00790.2012 23019314

[B108] Roth S. M. Ferrell R. E. Peters D. G. Metter E. J. Hurley B. F. Rogers M. A. (2002). Influence of age, sex, and strength training on human muscle gene expression determined by microarray. Physiol. Genomics 10, 181–190 10.1152/physiolgenomics.00028.2002 12209020 PMC2812433

[B109] Russ D. W. Gregg-Cornell K. Conaway M. J. Clark B. C. (2012). Evolving concepts on the age-related changes in “muscle quality.” J. Cachexia Sarcopenia Muscle 3, 95–109 10.1007/s13539-011-0054-2 22476917 PMC3374023

[B110] Ryushi T. Fukunaga T. (1986). Influence of subtypes of fast-twitch fibers on isokinetic strength in untrained men. Int. J. Sports Med. 7, 250–253 10.1055/s-2008-1025769 3793332

[B111] Satta A. Migliori G. B. Spanevello A. Neri M. Bottinelli R. Canepari M. (1997). Fibre types in skeletal muscles of chronic obstructive pulmonary disease patients related to respiratory function and exercise tolerance. Eur. Respir. J. 10, 2853–2860 10.1183/09031936.97.10122853 9493673

[B112] Schaufelberger M. Eriksson B. O. Grimby G. Held P. Swedberg K. (1995). Skeletal muscle fiber composition and capillarization in patients with chronic heart failure: relation to exercise capacity and central hemodynamics. J. Card. Fail. 1, 267–272 10.1016/1071-9164(95)90001-2 9420659

[B113] Schaufelberger M. Eriksson B. O. Grimby G. Held P. Swedberg K. (1997). Skeletal muscle alterations in patients with chronic heart failure. Eur. Heart J. 18, 971–980 10.1093/oxfordjournals.eurheartj.a015386 9183589

[B114] Stephens N. A. Gray C. Macdonald A. J. Tan B. H. Gallagher I. J. Skipworth R. J. (2012). Sexual dimorphism modulates the impact of cancer cachexia on lower limb muscle mass and function. Clin. Nutr. 31, 499–505 10.1016/j.clnu.2011.12.008 22296872

[B115] Stubbings A. K. Moore A. J. Dusmet M. Goldstraw P. West T. G. Polkey M. I. (2008). Physiological properties of human diaphragm muscle fibres and the effect of chronic obstructive pulmonary disease. J. Physiol. 586, 2637–2650 10.1113/jphysiol.2007.149799 18372305 PMC2464347

[B116] Suetta C. Aagaard P. Magnusson S. P. Andersen L. L. Sipila S. Rosted A. (2007). Muscle size, neuromuscular activation, and rapid force characteristics in elderly men and women: effects of unilateral long-term disuse due to hip-osteoarthritis. J. Appl. Physiol. (1985) 102, 942–948 10.1152/japplphysiol.00067.2006 17122381

[B117] Suetta C. Frandsen U. Jensen L. Jensen M. M. Jespersen J. G. Hvid L. G. (2012). Aging affects the transcriptional regulation of human skeletal muscle disuse atrophy. PLoS ONE 7:e51238 10.1371/journal.pone.0051238 23284670 PMC3526599

[B118] Sugiura S. Kobayakawa N. Fujita H. Yamashita H. Momomura S. Chaen S. (1998). Comparison of unitary displacements and forces between 2 cardiac myosin isoforms by the optical trap technique: molecular basis for cardiac adaptation. Circ. Res. 82, 1029–1034 10.1161/01.RES.82.10.1029 9622155

[B119] Sullivan M. J. Duscha B. D. Klitgaard H. Kraus W. E. Cobb F. R. Saltin B. (1997). Altered expression of myosin heavy chain in human skeletal muscle in chronic heart failure. Med. Sci. Sports Exerc. 29, 860–866 10.1097/00005768-199707000-00004 9243484

[B120] Sullivan M. J. Green H. J. Cobb F. R. (1990). Skeletal muscle biochemistry and histology in ambulatory patients with long-term heart failure. Circulation 81, 518–527 10.1161/01.CIR.81.2.518 2297859

[B121] Szentesi P. Bekedam M. A. Van Beek-Harmsen B. J. Van Der Laarse W. J. Zaremba R. Boonstra A. (2005). Depression of force production and ATPase activity in different types of human skeletal muscle fibers from patients with chronic heart failure. J. Appl. Physiol. (1985) 99, 2189–2195 10.1152/japplphysiol.00542.2005 16051711

[B122] Tanner B. C. McNabb M. Palmer B. M. Toth M. J. Miller M. S. (2014). Random myosin loss along thick-filaments increases myosin attachment time and the proportion of bound myosin heads to mitigate force decline in skeletal muscle. Arch. Biochem. Biophys. 552–553, 117–127 10.1016/j.abb.2014.01.015 24486373 PMC4043927

[B123] Taskin S. Stumpf V. I. Bachmann J. Weber C. Martignoni M. E. Friedrich O. (2014). Motor protein function in skeletal abdominal muscle of cachectic cancer patients. J. Cell. Mol. Med. 18, 69–79 10.1111/jcmm.12165 24251822 PMC3916119

[B124] Thedinga E. Karim N. Kraft T. Brenner B. (1999). A single-fiber *in vitro* motility assay. *in vitro* sliding velocity of F-actin vs. unloaded shortening velocity in skinned muscle fibers. J. Muscle Res. Cell Motil. 20, 785–796 10.1023/A:1005658825375 10730581

[B125] Thorstensson A. Larsson L. Tesch P. Karlsson J. (1977). Muscle strength and fiber composition in athletes and sedentary men. Med. Sci. Sports 9, 26–30 870781

[B126] Tikunov B. A. Mancini D. Levine S. (1996). Changes in myofibrillar protein composition of human diaphragm elicited by congestive heart failure. J. Mol. Cell. Cardiol. 28, 2537–2541 10.1006/jmcc.1996.0245 9004169

[B127] Toth M. J. Gottlieb S. S. Goran M. I. Fisher M. L. Poehlman E. T. (1997). Daily energy expenditure in free-living heart failure patients. Am. J. Physiol. 272, E469–E475 9124554 10.1152/ajpendo.1997.272.3.E469

[B128] Toth M. J. Matthews D. E. Ades P. A. Tischler M. D. Vanburen P. Previs M. (2005). Skeletal muscle myofibrillar protein metabolism in heart failure: relationship to immune activation and functional capacity. Am. J. Physiol. Endocrinol. Metab. 288, E685–E692 10.1152/ajpendo.00444.2004 15562248

[B129] Toth M. J. Miller M. S. Callahan D. M. Sweeny A. P. Nunez I. Grunberg S. M. (2013). Molecular mechanisms underlying skeletal muscle weakness in human cancer: reduced myosin-actin cross-bridge formation and kinetics. J. Appl. Physiol. (1985) 114, 858–868 10.1152/japplphysiol.01474.2012 23412895 PMC3633441

[B130] Toth M. J. Miller M. S. Vanburen P. Bedrin N. G. Lewinter M. M. Ades P. A. (2012). Resistance training alters skeletal muscle structure and function in human heart failure: effects at the tissue, cellular and molecular levels. J. Physiol. 590, 1243–1259 10.1113/jphysiol.2011.219659 22199163 PMC3381828

[B131] Toth M. J. Shaw A. O. Miller M. S. Vanburen P. Lewinter M. M. Maughan D. W. (2010). Reduced knee extensor function in heart failure is not explained by inactivity. Int. J. Cardiol. 143, 276–282 10.1016/j.ijcard.2009.02.040 19327849 PMC3411851

[B132] Trappe S. Gallagher P. Harber M. Carrithers J. Fluckey J. Trappe T. (2003). Single muscle fibre contractile properties in young and old men and women. J. Physiol. 552, 47–58 10.1113/jphysiol.2003.044966 12837929 PMC2343327

[B133] Trappe S. Godard M. Gallagher P. Carroll C. Rowden G. Porter D. (2001). Resistance training improves single muscle fiber contractile function in older women. Am. J. Physiol. Cell Physiol. 281, C398–C406 11443039 10.1152/ajpcell.2001.281.2.C398

[B134] Trappe S. Williamson D. Godard M. Porter D. Rowden G. Costill D. (2000). Effect of resistance training on single muscle fiber contractile function in older men. J. Appl. Physiol. (1985) 89, 143–152 10904046 10.1152/jappl.2000.89.1.143

[B135] Van Den Borst B. Koster A. Yu B. Gosker H. R. Meibohm B. Bauer D. C. (2011). Is age-related decline in lean mass and physical function accelerated by obstructive lung disease or smoking? Thorax 66, 961–969 10.1136/thoraxjnl-2011-200010 21724748 PMC3285455

[B136] Van Hees H. W. Van Der Heijden H. F. Ottenheijm C. A. Heunks L. M. Pigmans C. J. Verheugt F. W. (2007). Diaphragm single-fiber weakness and loss of myosin in congestive heart failure rats. Am. J. Physiol. Heart Circ. Physiol. 293, H819–H828 10.1152/ajpheart.00085.2007 17449557

[B137] Vescovo G. Dalla Libera L. Serafini F. Leprotti C. Facchin L. Volterrani M. (1998). Improved exercise tolerance after losartan and enalapril in heart failure: correlation with changes in skeletal muscle myosin heavy chain composition. Circulation 98, 1742–1749 10.1161/01.CIR.98.17.1742 9788828

[B138] Vescovo G. Serafini F. Facchin L. Tenderini P. Carraro U. Dalla Libera L. (1996). Specific changes in skeletal muscle myosin heavy chain composition in cardiac failure: differences compared with disuse atrophy as assessed on microbiopsies by high resolution electrophoresis. Heart 76, 337–343 10.1136/hrt.76.4.337 8983681 PMC484546

[B139] Walcott S. Warshaw D. M. Debold E. P. (2012). Mechanical coupling between myosin molecules causes differences between ensemble and single-molecule measurements. Biophys. J. 103, 501–510 10.1016/j.bpj.2012.06.031 22947866 PMC3414898

[B140] Wall B. T. Dirks M. L. Snijders T. Senden J. M. Dolmans J. Van Loon L. J. (2014). Substantial skeletal muscle loss occurs during only 5 days of disuse. Acta Physiol. (Oxf.) 210, 600–611 10.1111/apha.12190 24168489

[B141] Wang Y. Tanner B. C. Lombardo A. T. Tremble S. M. Maughan D. W. Vanburen P. (2013). Cardiac myosin isoforms exhibit differential rates of MgADP release and MgATP binding detected by myocardial viscoelasticity. J. Mol. Cell. Cardiol. 54, 1–8 10.1016/j.yjmcc.2012.10.010 23123290 PMC3535576

[B142] Watz H. Pitta F. Rochester C. Garcia-Aymerich J. Zuwallack R. Troosters T. (in press). An official ERS statement on physical activity in chronic obstructive pulmonary disease. Eur. Respir. J.

[B143] Weber M. A. Krakowski-Roosen H. Schroder L. Kinscherf R. Krix M. Kopp-Schneider A. (2009). Morphology, metabolism, microcirculation, and strength of skeletal muscles in cancer-related cachexia. Acta Oncol. 48, 116–124 10.1080/02841860802130001 18607877

[B144] Wenderoth M. P. Eisenberg B. R. (1987). Incorporation of nascent myosin heavy chains into thick filaments of cardiac myocytes in thyroid-treated rabbits. J. Cell Biol. 105, 2771–2780 10.1083/jcb.105.6.2771 3320054 PMC2114681

[B145] Whittom F. Jobin J. Simard P. M. Leblanc P. Simard C. Bernard S. (1998). Histochemical and morphological characteristics of the vastus lateralis muscle in patients with chronic obstructive pulmonary disease. Med. Sci. Sports Exerc. 30, 1467–1474 10.1097/00005768-199810000-00001 9789845

[B146] Widrick J. J. Knuth S. T. Norenberg K. M. Romatowski J. G. Bain J. L. Riley D. A. (1999). Effect of a 17 day spaceflight on contractile properties of human soleus muscle fibres. J. Physiol. 516(pt 3), 915–930 10.1111/j.1469-7793.1999.0915u.x 10200437 PMC2269300

[B147] Wilson G. J. Dos Remedios C. G. Stephenson D. G. Williams D. A. (1991). Effects of sulphydryl modification on skinned rat skeletal muscle fibres using 5,5′-dithiobis(2-nitrobenzoic acid). J. Physiol. 437, 409–430 1890642 10.1113/jphysiol.1991.sp018603 PMC1180055

[B148] Yu F. Hedstrom M. Cristea A. Dalen N. Larsson L. (2007). Effects of ageing and gender on contractile properties in human skeletal muscle and single fibres. Acta Physiol. (Oxf.) 190, 229–241 10.1111/j.1748-1716.2007.01699.x 17581136

[B149] Zizola C. Schulze P. C. (2013). Metabolic and structural impairment of skeletal muscle in heart failure. Heart Fail. Rev. 18, 623–630 10.1007/s10741-012-9353-8 23065040 PMC3784612

